# Genomic analysis of the rhesus macaque (*Macaca mulatta*) and the cynomolgus macaque (*Macaca fascicularis*) uncover polygenic signatures of reinforcement speciation

**DOI:** 10.1002/ece3.10571

**Published:** 2023-10-15

**Authors:** Nick Bailey, Cody Ruiz, Anthony Tosi, Laurie Stevison

**Affiliations:** ^1^ Department of Biological Sciences Auburn University Auburn Alabama USA; ^2^ Department of Anthropology Kent State University Kent Ohio USA

**Keywords:** genomics, macaques, reinforcement, speciation

## Abstract

Speciation can involve phases of divergent adaptation in allopatry and ecological/reproductive character displacement in sympatry or parapatry. Reproductive character displacement can result as a means of preventing hybridization, a process known as reinforcement speciation. In this study, we use whole‐genome sequencing (WGS) of two closely related primate species that have experienced introgression in their history, the rhesus (*Macaca mulatta*) and cynomolgus (*M. fascicularis*) macaques, to identify genes exhibiting reproductive character displacement and other patterns consistent with reinforcement speciation. Using windowed scans of various population genetic statistics to identify signatures of reinforcement, we find 184 candidate genes associated with a variety of functions, including an overrepresentation of multiple neurological functions and several genes involved in sexual development and gametogenesis. These results are consistent with a variety of genes acting in a reinforcement process between these species. We also find signatures of introgression of the Y‐chromosome that confirm previous studies suggesting male‐driven introgression of *M. mulatta* into *M. fascicularis* populations. This study uses WGS to find evidence of the process of reinforcement in primates that have medical and conservation relevance.

## Graphical abstract

In this study, we use whole‐genome sequencing (WGS) of two closely related primate species that have experienced introgression in their history, the rhesus (*Macaca mulatta*) and cynomolgus (*Macaca fascicularis*) macaques, to identify genes exhibiting a pattern of reproductive character displacement consistent with reinforcement speciation. Using windowed scans of various population genetic statistics to identify reproductive character displacement, we find 184 candidate genes associated with a variety of functions, including an overrepresentation of multiple neurological functions and several genes involved in sexual development and gametogenesis. This study is among the first to utilize WGS to analyze the phenomenon of reinforcement speciation and among the first to find evidence of this phenomenon in primates, particularly ones that have medical relevance.

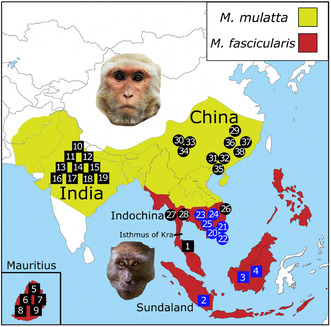

## 1 INTRODUCTION

Speciation is a crucial mechanism in generating biodiversity because it creates discontinuity between populations (Dobzhansky, 1937). In sexually reproducing species, especially animals, it is generally accepted that incipient species predominantly have a phase where they are allopatric (geographically separate) so that they can differentiate without gene flow (Coyne & Orr, 2004), but see Feder et al. (2012) for an alternate view. In allopatry, natural selection is likely to accelerate differentiation between populations due either to divergent selection for different ecological pressures (Nosil, 2012) or, as appears more often the case in vertebrates, selection in response to the same ecological pressures (Anderson & Weir, 2022). However, there are other mechanisms, namely reinforcement speciation, that can take place between parapatric (geographically bordering) or sympatric (geographically overlapping) populations and involve selective mechanisms other than ecological adaptation. Additionally, these processes are not mutually exclusive and can occur in varying degrees throughout the history of diverging populations (Baiz et al., 2019).

Reinforcement speciation is a process whereby hybridization between incipient species is selected against such that population fusion is prevented and the populations continue to diverge. For reinforcement to occur, hybridization must incur some fitness cost (suggesting an initial period of allopatry to allow divergence, followed by a period of parapatry or sympatry to allow for hybridization). Then some trait(s) that prevent hybridization must be selected for in one or both of the populations (Coyne & Orr, 2004; Pfennig & Pfennig, 2012; Rosenthal, 2017; Servedio & Noor, 2003). Reinforcement theory predicts a pattern of greater prezygotic isolation between sympatric/parapatric populations than between allopatric populations. This pattern of “reproductive character displacement” has been observed in amphibians, birds, fish, insects, mammals (including rodents and primates), plants, and reptiles (Allen et al., 2014; Coyne & Orr, 1989, 1997; Hopkins et al., 2012; Howard, 1993; Hubbs & Delco, 1960; Lemmon & Lemmon, 2010; Ortiz‐Barrientos et al., 2004; Smadja et al., 2015). Many past studies (including several cited above) examined this signature in morphological traits. However, this pattern can be analyzed using genomic data. By comparing estimates of divergence between individuals sequenced both from parapatry and allopatry (outside of the contact zone), one may test for the genomic signatures expected to be left by reinforcement (Garner et al., 2018). Generally, allopatric populations are expected to exhibit greater divergence because they are not undergoing hybridization. But if reinforcement is driving speciation, there should be regions of the genome that exhibit greater divergence between parapatric populations than allopatric ones, representing a signature of reproductive character displacement. Additionally, because reinforcement is a process of selection for traits that reduce hybridization, regions of the genome involved in reinforcement should exhibit directional selection and reduced introgression. Indeed, genetic evidence of reinforcement has been found in *Mus musculus* using microsatellite data (Smadja et al., 2015) and *Anolis* lizards using amplified fragment length polymorphism (Lambert et al., 2013). More recent studies have implemented larger‐scale genomic data to find evidence of reinforcement. Examples of these include analysis of SNP and indel data using ddRADseq in *Alouatta* monkeys (Baiz et al., 2019), analysis of CNV data using Pool‐seq in *Mus musculus* (North et al., 2020), and analysis of gene expression using whole‐transcriptome sequencing in *Pseudacris* frogs (Ospina et al., 2021).

**FIGURE 1:**
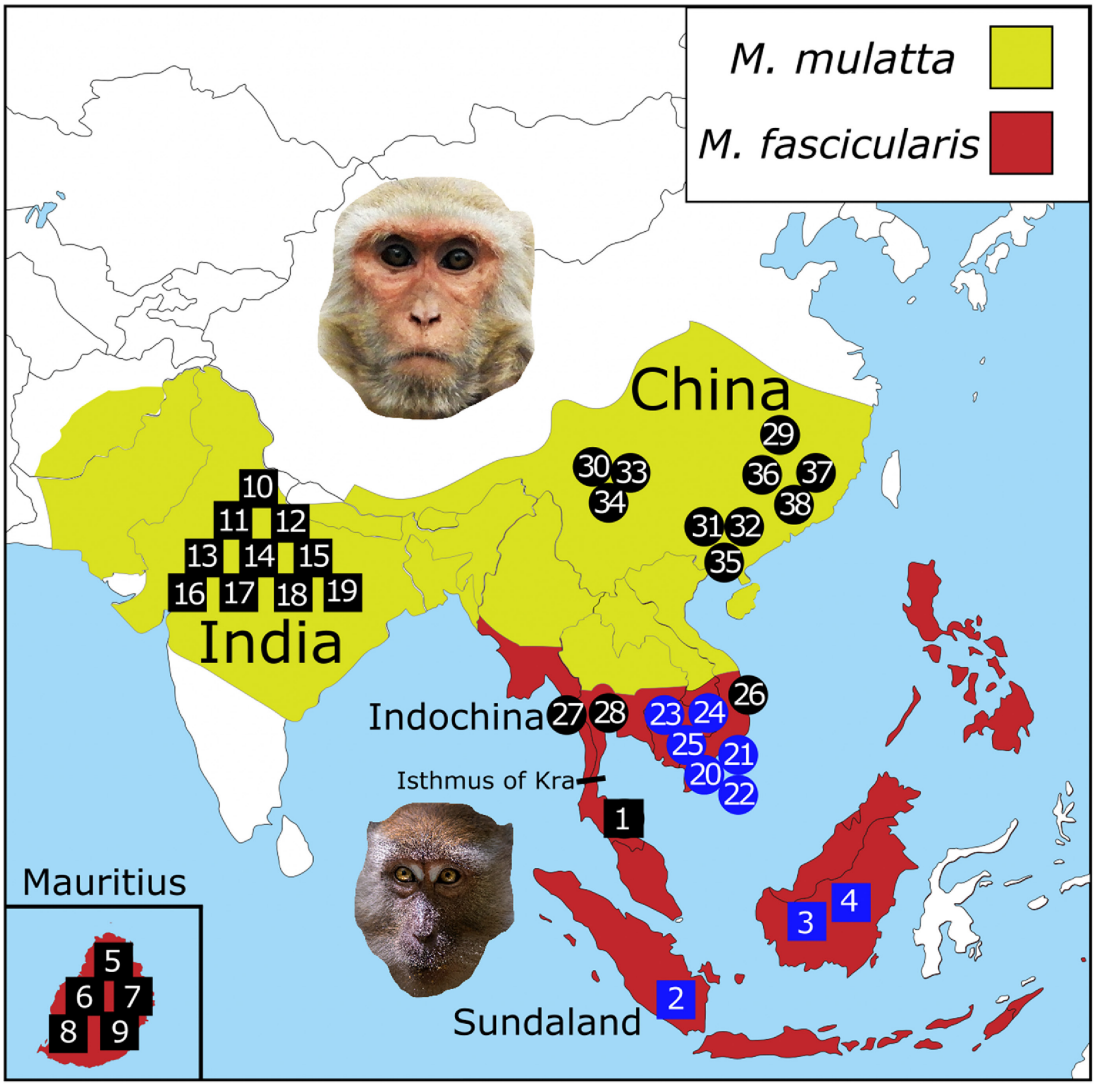
Range map of *Macaca mulatta* and *Macaca fascicularis* with samples in this study. Ranges (shaded in yellow for *M. mulatta* and red for *M. fascicularis*) were traced from IUCN Red List range maps last evaluated on November 20, 2015 (*M. mulatta*) and March 7, 2022 (*M. fascicularis*). Squares represent samples considered allopatric in this study, and circles represent parapatric samples. Numbers indicate approximate geographical origins of samples in Table 1. Blue shapes indicate sequences introduced in this study, and black shapes indicate previously sequenced samples. The sample numbers correspond to the order in which they appear in Table 1. The map was downloaded from https://d‐maps.com/carte.php?num_car=32142&lang=en. The top photo is of an *M. mulatta* individual from the National Zoological Park in Delhi, India, taken by Rajiv Bajaj. The bottom photo is of an *M. fascicularis* individual from Tanjung Puting National Park in Indonesia, taken by Dimitry B. Both were available through Unsplash.

In this study, we examine the possibility of reinforcement speciation between the rhesus macaque (*Macaca mulatta*) and the cynomolgus macaque (*M. fascicularis*) using whole‐genome sequencing. These species are the most widespread nonhuman primates, both due to their human commensalism (Small, 1998; Southwick & Siddiqi, 1998) and generalist ecology in terms of habitat and diet (Cui et al., 2019; Ross, 1992). Their ranges are parapatric to one another, with *M. mulatta* ranging through China and India, whereas *M. fascicularis* ranges throughout Indochina and Sundaland (biogeographic region including the Malay Peninsula and Indonesia), with introduced populations in Mauritius sourced from Sumatra (Tosi & Coke, 2007; Figure 1). The Isthmus of Kra in Thailand appears to be a significant biogeographic barrier in this system (Stevison & Kohn, 2009; Tosi et al., 2002), though it does not seem to preclude introgression completely (Bunlungsup et al., 2017; Osada et al., 2010). Notably, Indian and Chinese *M. mulatta* are separated from each other by the Himalaya mountains, while Indochinese and insular *M. fascicularis* are separated from each other by the strait of Malacca and a portion of the Pacific Ocean, in addition to the Isthmus of Kra (Zinner et al., 2013).

There are several lines of evidence to suggest that interactions between *M. mulatta* and *M. fascicularis* are characterized by reinforcement. It has been shown that gene flow across the contact zone between these species is asymmetric, predominantly with alleles from *M. mulatta* introgressing into *M. fascicularis* (Ruiz et al., 2021; Stevison & Kohn, 2009; Tosi et al., 2002). Y‐chromosome data from these same studies suggest male‐mediated introgression in particular, as Chinese *M. mulatta* and Indochinese *M. fascicularis* share several alleles on this chromosome with each other, to the exclusion of Indian *M. mulatta* and Sundaic *M. fascicularis*. This is in accordance with known female philopatry in macaques (Lawson Handley & Perrin, 2007). Thus, within the contact zone near the Isthmus of Kra, males of *M. fascicularis* may have to compete with the considerably larger males of *M. mulatta* (Zinner et al., 2013) for female *M. fascicularis* mates. Further, males of *M. fascicularis* from Indochina exhibit higher levels of aggression relative to Indonesian *M. fascicularis* recently transferred to a semi‐natural enclosure (Brent & Veira, 2001). In addition, infant *M. mulatta* from Indian‐Chinese crosses exhibited more irritable and aggressive behavior than Indian infants, and adult Chinese *M. mulatta* are known to be more aggressive in lab conditions than adult Indian *M. mulatta* (Champoux et al., 1997). One possibility that we hypothesize is that increased aggressiveness by individuals sourced from populations near the contact zone is a behavior selected by reinforcement to prevent interspecific mating, where we expect the signature to be stronger in parapatric *M. fascicularis*. We expect this because introgression is primarily from *M. mulatta* to *M. fascicularis* and not the reverse direction, so there should be more selection pressure on *M. fascicularis* to avoid heterospecific mating. Indeed, in most documented cases of heterospecific mating interactions, the fitness cost is greater in one species compared to the other (Gröning & Hochkirch, 2008). Another possibility that we hypothesize is that the aggressive behavior is introgressed from *M. mulatta* to *M. fascicularis*, which would only take place near the contact zone. In addition, it is known that *M. mulatta* sexually select for color traits not exhibited in *M. fascicularis*, such as red facial coloring (Dubuc, Allen, et al., 2014; Higham et al., 2021), suggesting the possibility of assortative mating for one's own species, which is usually expected to coincide with reinforcement (Servedio, 2000). However, it is worth noting that assortative mating is more likely to lead to reinforcement when introgression between populations is symmetrical (“two‐island model”) than in a scenario where introgression is asymmetrical (“continent‐island”; Servedio, 2000), where, as stated above, the latter more closely corresponds to the scenario examined in this study. Regardless, assortative mating can lead to reinforcement in either scenario (Servedio, 2000). Reinforcement is also only expected to act if there is a negative consequence to hybridization (e.g., inviability or sterility of offspring). It has been found that *M. fascicularis* within the parapatric zone exhibits reduced fecundity compared to Sundaic macaques (Kumpai et al., 2022).

*M. mulatta* and *M. fascicularis* are kept in captivity throughout China and the U.S.A. for medical research purposes (Feister, 2018; Zhang et al., 2017), resulting in a surplus of publicly available genomes. However, most *M. fascicularis* genome sequences are from an introduced population with low genetic diversity, that is, Mauritius. Here, we sequenced nine new *M. fascicularis* whole‐genome sequences representing populations from natural ranges in Indonesia, Vietnam, and Cambodia. A previous study of hybridization between these species (Jadejaroen et al., 2015) suggested that only regions south of China can be considered a true contact zone in the present day. That study found substantial differences in morphology between Chinese *M. mulatta* and Indochinese *M. mulatta*, where the latter exhibited more similarity to *M. fascicularis* than the former did. Thus, treating Chinese *M. mulatta* as parapatric in this study could allow for Chinese individuals that are far from the contact zone. However, a benefit of this approach is that our study should not include recent hybrids. This would cause difficulty classifying individuals as *M. mulatta* or *M. fascicularis*. Additionally, individuals with recent hybrid ancestry would necessarily represent a select group that may not exhibit signatures of past reinforcement since reinforcement reduces hybridization. Nonetheless, our study shows that there is detectable admixture between Chinese *M. fascicularis* and Indochinese *M. mulatta* (Figure 3) in accordance with previous studies using samples from these localities (Ruiz et al., 2021; Stevison & Kohn, 2009; Tosi et al., 2002), suggesting a wider range of overlap in the past or extensive introgression across the range mediated by hybridization in the contact zone.

Here, we test for signatures of reproductive character displacement between two species of macaques by estimating whole‐genome divergence landscapes from parapatric and allopatric populations of both species. However, reinforcement and reproductive character displacement are not always co‐occurring processes (Servedio, 2004). Therefore, we not only identify genomic regions with this signature of character displacement but also reduced introgression between parapatric populations (i.e., genomic regions where hybridization may be prevented when they introgress), as well as evidence for directional selection within parapatric *M. fascicularis* in exclusion of other populations (as this is the population primarily experiencing introgression, though all populations likely have other selective pressures in their own environments). Of the candidate genes generated by this approach, we focus on genes potentially involved in social behavior and reproduction as proxies of reproductive character displacement in particular as opposed to ecological character displacement (though we acknowledge it is difficult to exclude the possibility of ecological character displacement completely as it can incidentally cause changes in mate recognition signals (Noor, 1999)). Our results suggest that of multiple genes (though a small subset of the genome as a whole) spread throughout the genome, exhibiting a signature of reproductive character displacement consistent with reinforcement speciation.

## 2 MATERIALS AND METHODS

### 2.1 Samples and genome sequencing

For this study, nine *M. fascicularis* whole blood samples with known provenience were obtained from Labcorp Drug Development, formerly Covance. The Obtaining of these samples was in accordance with the Institutional Animal Care and Use Committee of Kent State University. Genomic DNA extracts were performed with a DNeasy Blood and Tissue Kit (QIAgen; Cat. No. 69504) on all whole blood samples, according to manufacturer guidelines. gDNA extracts were pair‐end sequenced on an Illumina NovaSeq 6000 platform through Novogene, producing 150‐bp reads across three lanes of sequencing. Additionally, 11 *M. fascicularis* and 20 *M. mulatta* genome samples were downloaded as FASTQ files (Cock et al., 2010) from NCBI SRA (Leinonen et al., 2011) using fasterq‐dump (sratoolkit version 2.10.8). Since reinforcement speciation is expected to have different effects on parapatric versus allopatric populations, we defined individuals as being part of one of four populations (allopatric *M. mulatta*, parapatric *M. mulatta*, allopatric *M. fascicularis*, and parapatric *M. fascicularis*). Respectively, these include (1) 10 *M. mulatta* from India (Yan et al., 2011 for one of those), (2) 10 *M. mulatta* from China, (3) five *M. fascicularis* from Mauritius, three from Indonesia (this study) and three from Thailand south of the Isthmus of Kra (Osada et al., 2020), and (4) four *M. fascicularis* from Vietnam (Yan et al. 2011 for one of those, three are from this study), three from Cambodia (this study), and two from a Chinese breeding farm, suggesting they are of Indochinese origin (Zhang et al., 2017). Data on these samples are provided in Table 1. Fastqc (version 0.11.9; Andrews, 2010) was conducted on all samples, and MultiQC (Ewels et al., 2016) was used to compile these results (see Data Availability Statement). Additional details on sequencing for all organisms (including read lengths and number of reads) are available in Table S1.

**TABLE 1:** Samples used in this study.

Locality	Sex	SRA number	Mapped	Duplicated	Final coverage	Homozygous	Heterozygous
Khao Noi/Khao Tangkuan, Songkhla, Thailand	Male	DRS139839	0.5946	0.0662	46.83	0.9976	0.0024
S. Sumatra, Indonesia	Male	SRR24738440	0.8395	0.3571	46.83	0.9978	0.0022
Indonesia	Female	SRR24738437	0.7452	0.1565	46.71	0.9978	0.0022
Indonesia	Female	SRR24738432	0.7554	0.1589	46.72	0.9979	0.0021
Mauritius	Male	SRS674436	0.7743	0.1405	46.93	0.9982	0.0018
Mauritius	Male	SRS674437	0.7753	0.1359	46.94	0.9981	0.0019
Mauritius	Male	SRS693707	0.7778	0.1356	46.95	0.9982	0.0018
Mauritius	Male	SRS693712	0.7679	0.1192	46.95	0.9982	0.0018
Mauritius	Male	SRS693714	0.7617	0.1457	46.95	0.9983	0.0017
India	Female	SRS2588651	0.69	0.1086	46.62	0.9985	0.0015
India	Female	SRS2588654	0.7161	0.1506	46.79	0.9984	0.0016
India	Female	SRS2588667	0.6864	0.1286	46.71	0.9984	0.0016
India	Male	SRS2588673	0.7027	0.1281	46.84	0.9984	0.0016
India	Female	SRS3755433	0.7486	0.1396	46.82	0.9984	0.0016
India	Female	SRS5035087	0.6653	0.1316	46.75	0.9984	0.0016
India	Female	SRS5035100	0.6639	0.1313	46.72	0.9985	0.0015
India	Female	SRS5035120	0.6604	0.1385	11.74	0.9986	0.0014
India	Male	SRS5035173	0.6678	0.1373	46.88	0.9984	0.0016
India	Male	SRS5035194	0.6729	0.1417	46.88	0.9984	0.0016
Dong Nai, Vietnam	Male	SRR24738439	0.7418	0.1573	46.89	0.9977	0.0023
Dong Nai, Vietnam	Male	SRR24738438	0.7731	0.1826	46.88	0.9976	0.0024
Dong Nai, Vietnam	Male	SRR24738436	0.7462	0.1593	46.92	0.9977	0.0023
Cambodia	Male	SRR24738435	0.736	0.1557	46.87	0.9976	0.0024
Cambodia	Male	SRR24738434	0.7585	0.1641	46.87	0.9976	0.0024
Cambodia	Male	SRR24738433	0.7495	0.1564	46.85	0.9976	0.0024
Vietnam	Female	SRS117874	0.4962	0.1591	46.89	0.9982	0.0018
Indochina (originally labeled as China)	Male	SRS4048144	0.7309	0.1945	46.84	0.9975	0.0025
Indochina (originally labeled as China)	Female	SRS4048145	0.742	0.2067	46.73	0.9978	0.0022
China	Female	SRS212016	0.4977	0.0655	46.57	0.9988	0.0012
Sichuan, China	Male	SRS748669	0.6659	0.125	46.74	0.9983	0.0017
Guangxi, China	Male	SRS778761	0.6715	0.118	46.78	0.9981	0.0019
Guangxi, China	Male	SRS791155	0.6677	0.1254	46.75	0.9981	0.0019
Sichuan, China	Male	SRS823980	0.6685	0.1191	46.73	0.9981	0.0019
Sichuan, China	Male	SRS823981	0.6658	0.1285	46.71	0.9982	0.0018
Guangxi, China	Male	SRS837317	0.6644	0.1151	46.75	0.9981	0.002
China	Female	SRS886329	0.7757	0.0995	46.58	0.998	0.002
China	Female	SRS886338	0.7602	0.1059	46.87	0.9979	0.0021
China	Female	SRS893058	0.6821	0.076	28.58	0.9981	0.002
Wat Paknam Pracharangsarith, Ranong, Thailand	Female	DRS139837	0.5855	0.0692	46.67	0.9992	0.0008
Mangrove Forest Research Center, Ranong, Thailand	Male	DRS139838	0.5854	0.0644	46.82	0.998	0.002

*Note*: The colors of rows represent geography and species of samples. Red = allopatric *M. fascicularis*, Green = allopatric *M. mulatta*, Blue = parapatric *M. fascicularis*, Purple = parapatric *M. mulatta*, White = no population assignment. The samples in white are included here since they went through our variant calling pipeline, but they were excluded from all analyses because they represent a subspecies (*Macaca fascicularis aurea*) that forms its own distinct cluster in PCA in exclusion of our assigned groups. The mapped column represents the proportion of reads mapped to the Mmul_10 (rheMac10) reference genome. The duplicated column represents the proportion of duplications identified prior to removal. Final coverage represents genome‐wide coverage depth after removing duplicates and base quality score recalibration. Homozygous and heterozygous columns represent the proportion of homozygous and heterozygous sites out of the total number that passed filtering. The order of samples corresponds to the number ID given in Figure 1.

### 2.2 Genomic pipeline for autosomes and sex chromosomes

The following steps describe the pipeline used for autosomes and largely follow the GATK Best Practices for calling germline variants from short‐read data (Van der Auwera & O'Connor, 2020). All samples were aligned to the *M. mulatta* reference genome rheMac10 (RefSeq ID: GCF_003339765.1), which was downloaded from the UCSC Genome Browser (Kent et al., 2002). This file was indexed using bwa (version 0.7.17; Li, 2013), SAMtools (version 1.11; Li et al., 2009), and picard CreateSequenceDictionary (GATK version 4.1.9.0). The alignment was conducted using bwa‐mem. GATK was then used to mark duplicates and conduct base quality score recalibration (BQSR) using known Indian *M. mulatta* variants from mGAP (Bimber et al., 2019) and a study of Chinese *M. mulatta* (Liu et al., 2018). These known variants were aligned to rheMac8, so we used GATK LiftOver VCF to convert coordinates to the rheMac10 reference. GATK was also used for variant calling. GATK Haplotype Caller was also used for variant calling (with 2 as ploidy). This subsequently involved the creation of a genomics database using GenomicsDBImport, and joint genotyping (with 2 as ploidy), a process of calling variants in all samples simultaneously to use maximal information from a cohort using GenotypeGVCFs. After this, invariant sites were filtered for a hard threshold where sites with a depth less than 5 and quality less than 20 were removed using BCFtools (version 1.11; Danecek et al., 2021). Variant sites were filtered using GATK variant quality score recalibration (VQSR; Figure S2) with a truth sensitivity filter of 99.0. Throughout the pipeline, SAMtools coverage and flagstat were used for quality control (before marking duplicates, after marking duplicates but before BQSR, and after BQSR to check mapping quality, depth, and coverage throughout). Files were also compressed for permanent storage and checked for data integrity using bam2cram_check (Colgiu, 2016) and genozip (version 13.0.11; Lan et al., 2021).

For X‐chromosomes in females, the above pipeline was used with the exception that chrY was removed from rheMac10 using seqkit (version 0.8.1; Shen et al., 2016). This was done to avoid incorrect mapping of chrX to PARs on chrY. Notably, the genome for rheMac10 was originally obtained from a female macaque with a Y‐chromosome BAC added in the version on NCBI (https://www.ncbi.nlm.nih.gov/data‐hub/genome/GCF_003339765.1/). This female reference FASTA was indexed, and female samples were aligned to it as above. After alignment, chrX was subset from the whole genome file to continue the pipeline as described above, except a genomic database separate from the autosomes was created prior to joint genotyping and all steps involving a reference FASTA using the rheMac10 female reference.

For X and Y chromosomes in males, the pipeline is as described for autosomes, except in the following respects. chrX and chrY were subsets after the alignment to continue through the pipeline. For haplotype calling and joint genotyping, ploidy was set at 1 (since in males, X and Y are effectively haploid), and prior to joint genotyping, the male X and Y chromosomes had unique genomic databases created separately.

### 2.3 Population structuring analyses

Principal component analysis was conducted using plink (version 2.00a3) on data from all 40 macaques described in Samples and Genome Sequencing. PCA was conducted on all autosomes together (random seed: 1674576807), the Y‐chromosome (random seed: 1674579062), and the X‐chromosomes separately for males (random seed: 1675285981) and females (random seed: 1675286037) to determine population structure. Males and females were analyzed separately in the PCA because the difference in ploidy for the X‐chromosome can affect the variances (Patterson et al., 2013). It was apparent that two of the Thailand macaques sampled were extreme outliers, grouping with only themselves to the exclusion of all other samples. These two are members of the *M. fascicularis* subspecies *aurea* (Osada et al., 2020) and may have introgressed with *sinica* group species distantly related to *M. mulatta* and *M. fascicularis* (Matsudaira et al., 2018). Therefore, these samples were removed from subsequent analysis and from the principal component analysis presented here (Figure 3). Tracy–Widom tests (Patterson et al., 2006) were conducted on the datasets to evaluate the statistical significance of different principal components using a pre‐compiled binary of EIGENSOFT 5.0 for Mac (Patterson et al., 2013). This test is unreliable when presented with too few eigenvalues, so plink was run to produce the maximum number of eigenvalues per analysis, which is the number of individuals subtracted by 1. Therefore, this study used inputs of 37 for autosomes, 22 for the Y‐chromosome, 22 for the X‐chromosome in males, and 14 for the X‐chromosome in females. We plotted our PCA results using a custom R script (see Data Availability Statement) based on a publicly available script (Mészáros, 2021).

In addition, we conducted ADMIXTURE (version 1.3.0; Alexander et al., 2009) analyses on all autosomes together and the Y‐chromosome. ADMIXTURE was conducted using *K* = 2, which likely corresponds to the two distinct species, instead of *K* = 4, which we may expect to correspond to parapatric and allopatric populations of both species. This is because *K* = 2 produces the lower cross‐validation score (0.39626) and because we wanted to examine *M. mulatta* ancestry within parapatric *M. fascicularis*. We plotted our ADMIXTURE results using a publicly available GitHub script (Meier, 2021).

### 2.4 Determining genomic window sizes

The software program GenWin (Beissinger et al., 2015) was used to determine appropriate window lengths for autosomes and X‐chromosomes in our dataset. The Y‐chromosome was only analyzed as a single unit and is not present in our candidate gene analyses. GenWin uses data for a given statistic (e.g., *F*_ST_) conducted per site to apply a smoothing spline and define window boundaries by inflection points. This allows for varying window lengths, ideally representing independently recombining and evolving regions of the genomes, to avoid non‐independent testing of windows. Beissinger et al. (2015) recommend using the same statistic to calculate the per site estimates and to calculate estimates over windows given by the per site smoothing spline. However, since we were interested in calculating multiple statistics over these windows, including Tajima's *D*, which cannot be calculated for a single site, we chose to conduct the smoothing spline analysis with a more fundamental statistic. Therefore, we calculated minor allele frequencies for all variable sites using BCFtools and input these data into GenWin to determine window sizes used for all subsequent statistics. The resulting windows average 10,679 bases in length, with a range of 53 to 1,886,200 bases and a standard deviation of 14,005 bases. The relatively small window length is likely due to extensive recombination that has occurred since the divergence of *M. mulatta* and *M. fascicularis*. The wide range is likely due to variations in recombination rates throughout the genome. The average is much closer to the minimum value of the range than the maximum, suggesting a positive‐skewed distribution.

### 2.5 Population genetic analysis

**FIGURE 2:**
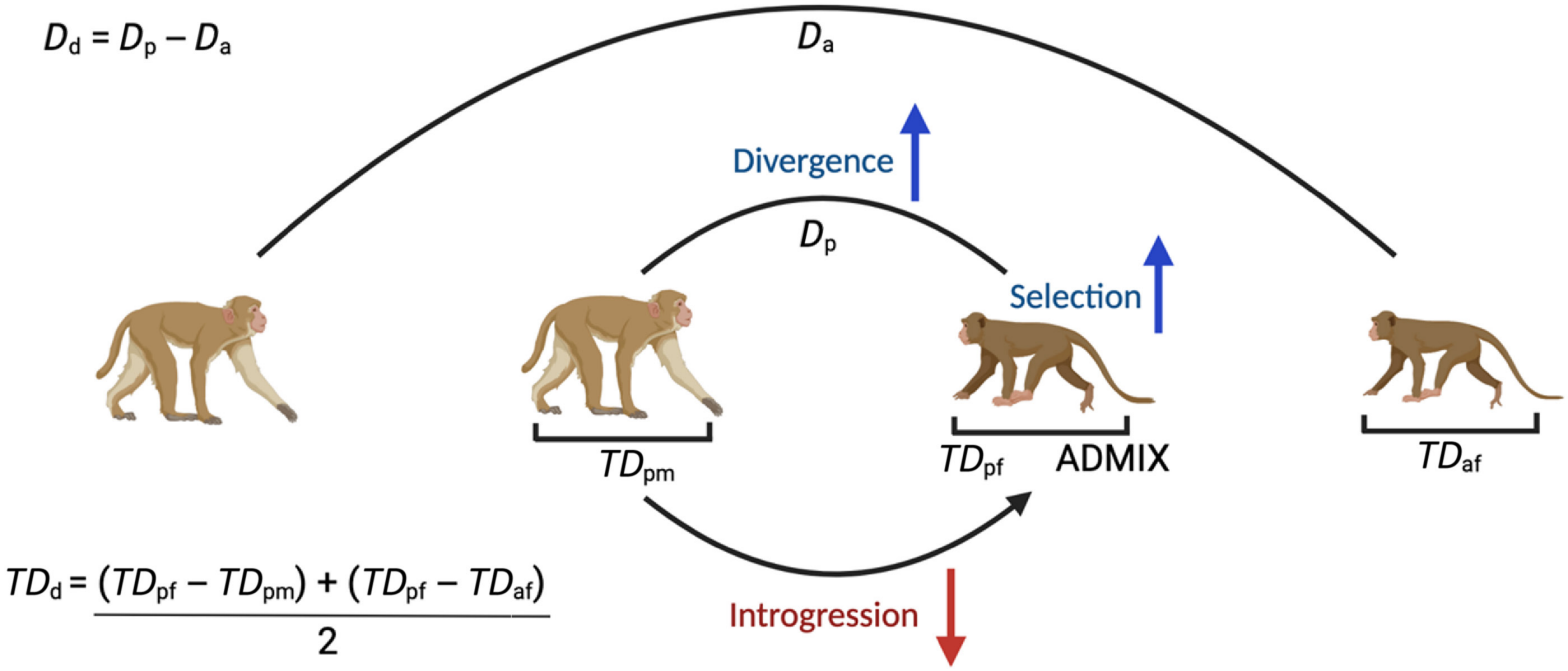
Conceptual figure of analyses applied in this study. From left to right, macaques represent allopatric *Macaca mulatta*, parapatric *M. mulatta*, parapatric *Macaca fascicularis*, and allopatric *M. fascicularis*. In a reinforcement scenario, we expect that some regions of the genome should exhibit increased divergence between parapatric populations (*D*_*p*_), increased selection in the population receiving introgression (*TD*_*pf*_), and reduced admixture in the population receiving introgression (ADMIX). Variable names in the figure represent variables described for Equations 1 and 2, which are given here as well. The image was created using BioRender.

Populations undergoing reinforcement are expected to have regions of the genome exhibiting high divergence in parapatry, divergent selection, and reduced introgression. Thus, we calculated *F*_ST_ and *D*_XY_ with pixy (version 1.2.6; Korunes & Samuk, 2021) to infer divergence, Tajima's *D* with scikit‐allel (version 1.3.3; Miles, 2015) to infer divergent selection, and ADMIXTURE (version 1.3.0; Alexander et al., 2009) to infer introgression (Figure 2). These statistics were calculated in windows defined by spline analysis with GenWin (see above Section 2.4). Based on previous studies (Stevison & Kohn, 2009) and our own whole‐genome ADMIXTURE analysis, introgression is predominantly from *M. mulatta* into *M. fascicularis*, so we expect parapatric *M. fascicularis* in particular to exhibit signatures of selection for reinforcement.

### 2.6 Candidate gene detection

Higher divergence, as estimated using *F*_ST_ and *D*_XY_, between parapatric populations of different species than allopatric populations is the characteristic signature of reproductive character displacement (Garner et al., 2018). Therefore, for each genomic window and for each divergence statistic, the difference between parapatric populations of *M. mulatta* and *M. fascicularis* and between allopatric populations of *M. mulatta* and *M. fascicularis* was calculated (not within species), as in the following formula:

(1) $$D_{p}-D_{a}=D_{d}$$

Here, *D*_*p*_ is divergence (either *F*_ST_ or *D*_XY_) calculated between parapatric populations, and *D*_*a*_ is divergence calculated between allopatric populations. *D*_*d*_ therefore represents the difference between these values, where genomic windows exhibiting character displacement are expected to be greater than 0 as divergence should be greater between parapatric populations than allopatric populations (Figure 2). Then, to generate a null distribution of the expected value of *D*_*d*_ for each window, 100 population files were generated where individuals were randomly assigned to one of the four populations to generate 100 permutation estimates. *D*_*d*_ for each window was considered significant when the empirical value was greater than 95% of the permutation values for the same window.

Tajima's *D* was calculated for parapatric *M. fascicularis*, parapatric *M. mulatta*, and allopatric *M. fascicularis*. Since we expected reinforcement to affect parapatric *M. fascicularis* most strongly, we looked for signatures of directional selection (negative Tajima's *D*) within this group only in exclusion of the hybridizing group (parapatric *M. mulatta*) and conspecifics (allopatric *M. fascicularis*), as these could share similar demographic and selective factors. Given that allopatric *M. mulatta* neither hybridizes with *M. fascicularis* nor is conspecific to them, comparison between them should not be necessary to generate a null expectation for parapatric *M. fascicularis*. Then, for each window, the averaged Tajima's *D* difference was calculated with the following formula:

(2) $$\frac{\left({\mathrm{TD}}_{\mathrm{pf}}-{\mathrm{TD}}_{\mathrm{pm}}\right)+\left({\mathrm{TD}}_{\mathrm{pf}}-{\mathrm{TD}}_{\mathrm{af}}\right)}{2}={\mathrm{TD}}_{d}$$

Here, *TD*_*pf*_ is the Tajima's *D* value in parapatric *M. fascicularis*, *TD*_*pm*_ is the Tajima's *D* value in parapatric *M. mulatta*, and *TD*_*af*_ is the Tajima's *D* value in allopatric *M. fascicularis*. The equation takes the difference of Tajima's *D* between parapatric *M. fascicularis* and each other group and then divides by 2 to average these differences. Therefore, *TD*_*d*_ is the averaged difference of Tajima's *D* for parapatric *M. fascicularis* (Figure 2). For this statistic, we considered values less than −1 as significant.

For ADMIXTURE, windows were extracted where the average proportion of *M. mulatta* ancestry in parapatric *M. fascicularis* was less than 2.5%, as it is expected that genomic regions exhibiting reinforcement should have low introgression between *M. mulatta* and *M. fascicularis*. The cutoff values for significance used for both Tajima's *D* and ADMIXTURE analyses are post‐hoc values chosen to make these statistics more stringent than our permutation method for *F*_st_/*D*_xy_ (see Section 3 for numbers of windows that resulted from these cutoffs).

Finally, candidate windows were then defined as ones that fulfill these criteria for Tajima's *D*, ADMIXTURE, and in either *F*_ST_ or *D*_XY_. Therefore, candidate windows should exhibit all three previously discussed signatures of reinforcement in this system: (1) increased divergence in parapatry between both species; (2) increased selection on the population that has been introgressed into (parapatric *M. fascicularis*); and (3) decreased introgression in this same population (Figure 2). The Bioconductor (Gentleman et al., 2004) GenomicRanges package (Lawrence et al., 2013) was used to intersect windows for all statistics and to find overlapping genes contained in the Ensembl gene annotation for rheMac10 (https://hgdownload.soe.ucsc.edu/goldenPath/rheMac10/bigZips/genes/rheMac10.ensGene.gtf.gz; Howe et al., 2021).

### 2.7 Candidate gene analysis

gProfiler (Raudvere et al., 2019), a website aggregating multiple functional databases, was used to conduct overrepresentation tests for Gene Ontology (GO), Kyoto Genes and Genomes (KEGG) pathways, and Human Phenotype (HP) databases using g:SCS multiple testing correction because this method has been shown to account for the hierarchical nature of GO terms better than methods such as Bonferonni correction and Benjamini–Hochberg FDR correction, which assume all tests are independent (Reimand et al., 2007). We then subset genes resulting from our analysis that matched terms of a priori interest for reinforcement speciation in this system. These terms were “estrogen,” “aggressive,” “melanogenesis,” “reproducti” (to include reproduction and reproductive), and “social behavior,” regardless of the overall representation of these terms in our dataset. We expected these phenomena to be relevant to reinforcement in this system in different ways. The estrogen signaling pathway contributes to sexual swelling in macaques (Dixson, 1998; Rhodes et al., 1997; Vandenbergh, 1965), and increased sexual skin redness and darkness are sexually selected in *M. mulatta* (Dubuc, Allen, et al., 2014; Dubuc, Winters, et al., 2014; Higham et al., 2021), so it may contribute to assortative mating in this system. Melanogenesis may also be relevant to this coloration, as it is involved in pigmentation in mammals broadly (Rosenthal, 2017). Both species exhibit increased aggressive behavior toward heterospecifics, so we hypothesized this may be a mechanism to prevent heterospecific crosses (Brent & Veira, 2001; Champoux et al., 1997). Parapatric *M. fascicularis* that have hybridized with *M. mulatta* have been showed to have reduced fecundity (Kumpai et al., 2022), suggesting some isolation related to reproductive development. Lastly, mating interactions are a broad subset of social behavior, so we expect terms in this category to be relevant as well (Rosenthal, 2017). The purpose of subsetting these categories was then do a literature search of these subset genes to examine their functions in more detail and confirm associations given in the GO, KEGG, and HP databases within gProfiler.

We subset VCFs for each gene using BCFtools, then combined these and then subset sites with at least one variant unique to parapatric *M. fascicularis* using a custom Python script (see Data Availability Statement). We annotated this “Parapatric *M. fascicularis* Unique” VCF with SnpEff (version 5.0; Cingolani et al., 2012) to identify putative functions of variants. We then further analyzed it with MEME suite's tomtom program (version 5.4.1; Bailey et al., 2009) to identify motif binding sites for transcription factors and SIFT4G (Vaser et al., 2016) to score missense variants. For the former, we used SnpSift to isolate sites identified as “intron_variant,” “upstream_gene_variant,” “downstream_gene_variant,” “5_prime_UTR_variant,” and “3_prime_UTR_variant” and place these in a “motif” VCF as these are all possible binding sites for transcription factors. For the latter, we used SnpSift to isolate “synonymous_variant” and “missense_variant” as these occur in coding regions.

Motif finding requires scanning a sequence (not a single site) against a position weight matrix (Stormo, 2015). Therefore, a conversion of VCF variants to FASTA sequences is necessary for using tomtom. For this reason, variant positions in the “motif” VCF were used to generate a bed file of 13‐bp sequences (6 nucleotides flanking the variant position on both sides) for each variant. We made a variant consensus rheMac10 genome FASTA using BCFtools consensus, which replaces all the VCF REF alleles in a FASTA genome with the VCF ALT alleles. We used BEDtools getfasta (version 2.30.0; Quinlan & Hall, 2010) to generate FASTA sequences of all the putative motif sequences using both the rheMac10 reference genome and the variant consensus genome. Since these sequences are based on sites where parapatric *M. fascicularis* had at least one unique variant, then the reference alleles should correspond to *M. mulatta* (given that the reference genome is this species), and the alternate alleles should correspond to *M. fascicularis*. Therefore, for identifying putative transcription factors in the reference sequences, we used a *M. mulatta* motif database, and for alternate sequences, we used a *M. fascicularis* motif database. We downloaded both databases from the MEME suite website, which were generated by CIS‐BP_2.00 (Weirauch et al., 2014) (Stormo, 2015). The rationale for using 13‐bp for the FASTA sequences is that this is the average length of motifs in both databases. We used this approach to identify putative transcription factors that are unique to *M. fascicularis* variants in the windows of interest and thus may be associated with variants experiencing reinforcement selection.

We scored missense variants using SIFT4G. SIFT uses information from sequence alignments and the chemical properties of amino acids to predict if missense mutations are deleterious or non‐deleterious (scored on a scale ranging from 0 to 1). The SIFT4G annotator (Vaser et al., 2016) was used to annotate the “parapatric *M. fascicularis* Unique” VCF with SIFT scores corresponding to the parapatric *M. fascicularis*‐derived alleles. For this purpose, we created a SIFT database for rheMac10. SIFT scores for the corresponding reference allele given in the rheMac10 database were subtracted from these values using a custom script (github repo) to generate a B‐SIFT score that can be used to infer deleterious, neutral, or adaptive mutations (this score ranges from −1 to 1; Lee et al., 2009). The authors of that paper used a cutoff of 0.5 or above to classify a mutation as adaptive though this resulted in low statistical sensitivity (9%) and very high specificity (99%). So in this study, we opted to classify any positive B‐SIFT score as an adaptive mutation after filtering out scores that received low confidence warnings from SIFT4G.

## 3 RESULTS

### 3.1 Genome sequencing and processing data

This study involved Illumina resequencing of *M. fascicularis* (*N* = 9) individuals and processing of previously sequenced *M. mulatta* (*N* = 20) and *M. fascicularis* (*N* = 11) samples. The alignment mapping to the reference genome ranged from 49.62% to 83.95% and averaged 70.08%, with a median of 70.94%. The final depth of sequencing coverage after removing duplicated reads ranged from 11.74 to 46.95× and averaged 45.47× with a median of 46.82×. The total number of SNPs identified after filtering was 31,639,415 bp out of a total of 1,333,185,598 sites kept after filtering, thus composing 2.37% of all sites. Per‐individual heterozygosity ranged from 0.08% to 0.25% of all unfiltered sites, with an average and median of 0.19% (Table 1). Other information, such as the number of reads and average read length, is available in Table S1.

### 3.2 Population structure analyses

**FIGURE 3:**
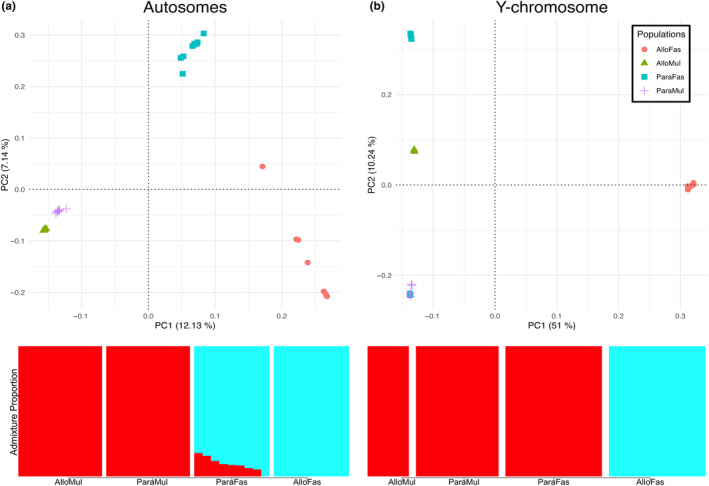
Principal component analysis and ADMIXTURE plots for samples in this study. (a) PCA and ADMIXTURE plot for all autosomes combined. (b) PCA and ADMIXTURE for Y‐chromosomes. Population labeling for all plots is as follows: AlloFas, Allopatric *M. fascicularis*; AlloMul, Allopatric *M. mulatta*; ParaFas, Parapatric *M. fascicularis*; ParaMul, Parapatric *M. mulatta*. In ADMIXTURE plots, the color red represents the proportion of *M. mulatta* ancestry, whereas the color cyan represents the proportion of *M. fascicularis* ancestry.

Principal component analysis for the autosomes included 30,874,358 sites and separated the four populations that were defined for this study along the first principal component (PC1), which accounts for 12.13% of the variance in the data (*p* = 1.77e‐14). Notably, along this axis, the allopatric populations of different species are the most distant from each other. PC2, which accounts for 7.14% of the variance in the data (*p* = 1.01e‐11), shows high genetic variability within and between the *M. fascicularis* populations compared to *M. mulatta* (Figure 3b). The third and fourth PCs are also statistically significant and, respectively, account for 4.66% (*p* = .005) and 4.41% (*p* = .002) of the variance in the data (Figure S1c). The former separates the parapatric and allopatric *M. mulatta* from each other, and the latter separates distinct subpopulations of the allopatric *M. fascicularis*, which exhibit variation within and between land masses (Scheffrahn et al., 1996). Our ADMIXTURE analysis on autosomes confirms the introgression of parapatric *M. mulatta* into parapatric *M. fascicularis*, with all except one of the latter individuals showing evidence of *M. mulatta* ancestry. The amount of *M. mulatta* ancestry in these individuals ranges from 0% to 18%. None of the *M. mulatta* individuals in our study themselves show evidence of *M. fascicularis* ancestry, which is in accord with the known unidirectional introgression of *M. mulatta* into *M. fascicularis* previously referenced.

The principal component analysis for the Y‐chromosomes included 30,984 sites and separated the allopatric *M. fascicularis* from all other samples along PC1, which accounts for 51% of the variance in the data. While this split is in accordance with the known patterns of Y‐introgression between these populations (Matsudaira et al., 2018; Stevison & Kohn, 2009; Tosi et al., 2002), this PC is marginally statistically significant (*p* = .049). Our ADMIXTURE analysis on the Y‐chromosome additionally confirms the swamping of parapatric *M. fascicularis* Y‐chromosomes with *M. mulatta* Y‐chromosomes, as there is no detectable *M. fascicularis* ancestry in our Y‐chromosome analysis for parapatric *M. fascicularis* individuals. PC2, which accounts for 10.24% of the variance in our data, separates parapatric *M. fascicularis* from parapatric *M. mulatta* (with the exception of two parapatric *M. fascicularis* grouping with *M. mulatta*) at the two extremes, with allopatric populations of both species being in the middle along this axis. Divergence between parapatric populations of different species but not allopatric populations is consistent with a pattern of character displacement on the Y‐chromosome, though this PC is marginally statistically insignificant (*p* = .077; Figure 3b).

The principal component analysis for the X‐chromosomes included 728,640 sites and gave similar results for both sexes. For both analyses, PC1 separates *M. mulatta* and *M. fascicularis*, PC2 separates both populations of *M. fascicularis*, and PC3 separates both populations of *M. mulatta*. The first two PC's for the male plot are statistically significant (PC1 *p* = 1.27e‐06; PC2 *p* = 3.91e‐08), while PC3 is marginally not (*p* = .072; Figure S1a). For the female plot, only PC1 is significant (*p* = .037; Figure S1b).

### 3.3 Windows and genes identified as candidates for reinforcement

**FIGURE 4:**
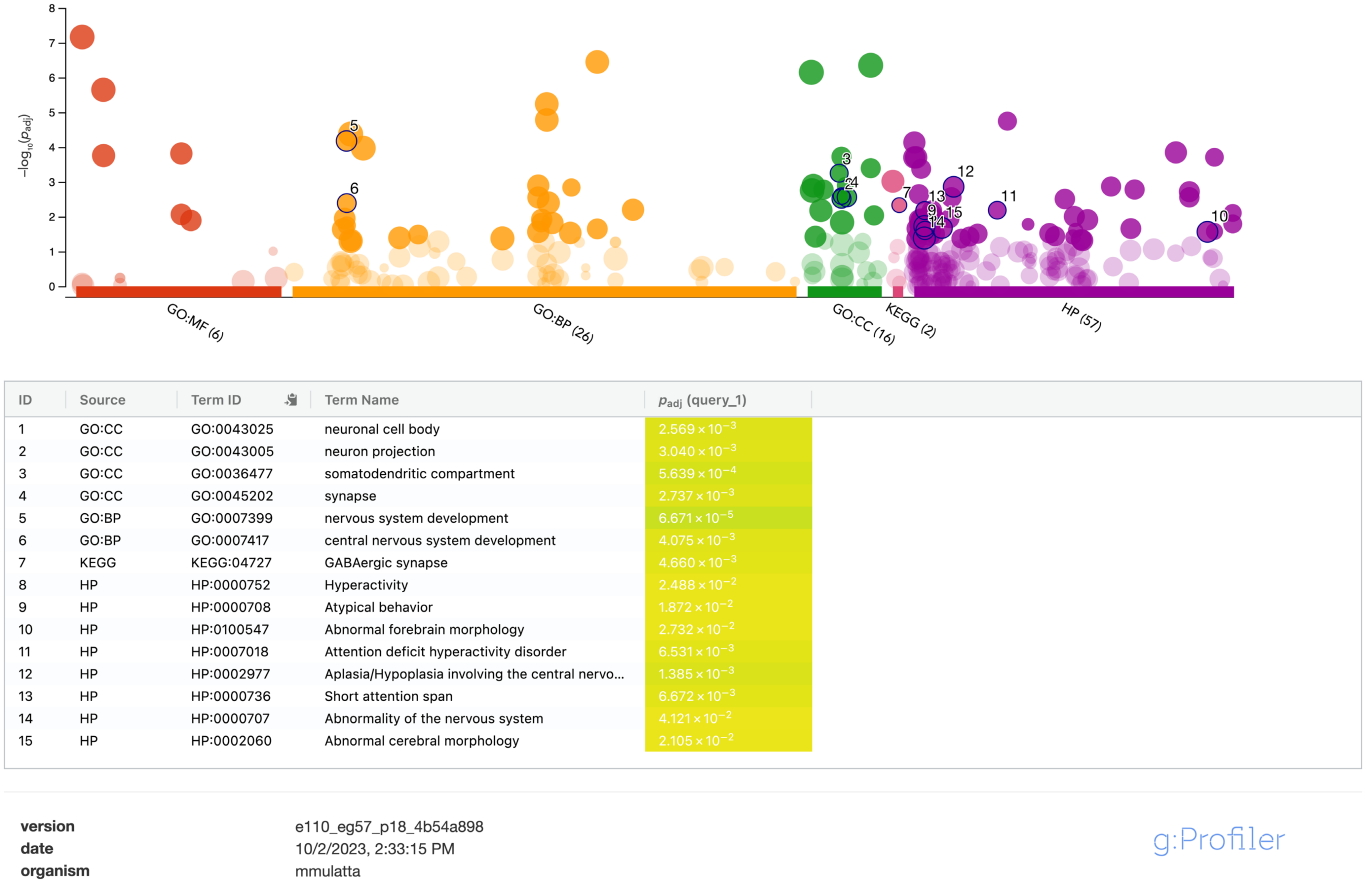
gProfiler results for the candidate gene list. Dot plot showing results of category overrepresentation tests of candidate genes compared to total genes annotated in the rheMac10 genome. Each dot represents a GO, KEGG, or HP category, and the *y*‐axis represents a negative log transform of the adjusted *p*‐value, such that higher dots are more statistically significant. Significant categories are opaque, and non‐significant categories are transparent. Categories outlined in black with numbers correspond to the neurological and behavioral categories depicted in the table below.

We identified 238 genomic windows that fulfilled our criteria for either *F*_ST_ or *D*_XY_, Tajima's *D*, and ADMIXTURE for candidate gene detection, all of which were autosomal, out of a total of 265,701 windows (0.08957%) examined. For each summary statistic individually, the following number of windows were considered significant: 14,089 for *F*_ST_; 19,662 for *D*_XY_; 12,864 for Tajima's *D*; 13,471 for ADMIXTURE. The 238 windows overlap 184 genes out of a total of 35,432 (0.5193%) annotated in the rheMac10 genome. A complete list of the 184 genes and categories examined by gProfiler can be found in Table S2 or accessed through the gProfiler website (see Data Availability Statement), though we note here that 86 GO/KEGG/HP terms were statistically overrepresented by the candidate genes in this study. Of particular interest here were a variety of neurological terms that were overrepresented, including “Behavioral abnormality,” “Attention deficit hyperactivity disorder,” “Hyperactivity,” “Short attention span,” “GABAergic synapse,” “somatodendritic compartment,” “neuronal cell body,” “dendrite,” “dendritic tree,” “neuron projection,” “central nervous system development,” and “nervous system development” (Figure 4). We also subset the following terms from the gProfiler results of this study to conduct a literature search on genes associated with them: “aggressive,” “reproducti,” “social behavior,” “estrogen,” and “melanogenesis” (see Section 2). From this literature search, the genes of potential interest we found in each of these categories were: reproduction (RARA, M1AP, TRIP13, RNF216, AMHR2, CLP1), social behavior (NPAS4), estrogen (RARA, ADCY2), and melanogenesis (GNAI2). We detail previous studies on these genes resulting from our literature search and their relevance as candidate reinforcement genes in the discussion. Notably, this study did not find any of the 40 previously identified candidate genes for aggressive behavior in mammals (Zhang‐James et al., 2019) in the candidate gene dataset, and all genes in this dataset annotated by gProfiler as involved in aggressive behavior could not be confirmed as such by a literature search. The term “aggressive behavior” is unique to the HP database and not present in the GO and KEGG annotations, suggesting this inability to corroborate the gProfiler annotation in our literature search is unique to this database.

### 3.4 Functional characterization of variants and motif analysis

Using a custom python script (see Data Availability Statement) to subset sites for the 184 candidate genes that contain at least one allele unique to the parapatric *M. fascicularis*, 54,550 sites were extracted. The putative functional changes of these mutations were characterized by SnpEff. Of these, two were nonsense variants, 392 were missense variants, 580 were synonymous coding variants, 51,197 were intron variants, 3726 were upstream gene variants, 3621 were downstream gene variants, 197 were 5′UTR variants, 590 were 3′UTR variants, and 110 were splice region variants (of which 1 and 2 were, respectively, splice acceptor and splice donor variants; see Data Availability Statement for SnpEff).

Tomtom motif analysis utilized 53,964 sites in intron, upstream, downstream, and UTR regions of the candidate genes. Of these, 14,048 had at least one predicted transcription factor unique to the alternate allele, which for these sites is unique to parapatric *M. fascicularis*.

B‐SIFT analysis utilized 472 sites in coding regions after filtering out sites with low confidence warnings from SIFT4G. These scores took the difference of the SIFT score for the alternate allele, which as above, is unique to parapatric *M. fascicularis*, from the SIFT score for the reference allele. Because a variant can affect multiple transcripts, this resulted in 1175 distinct B‐SIFT scores. Of these, 850 were predicted neutral (score of 0), 282 were predicted deleterious (negative score), and 43 were predicted adaptive (positive score). A list of variants and associated genes can be found in Table S2.

## 4 DISCUSSION

### 4.1 Genetic diversity of *M. fascicularis*

As part of this study, we sequenced nine genomes of *M. fascicularis* individuals sourced from mainland and insular southeast Asia. An in‐house survey of published *M. fascicularis* genomes revealed that 81% of those available from NCBI are from Mauritius, a small island off the coast of Africa where this species has been introduced by humans. While their low genetic diversity makes this isolated group suitable for biomedical research, these samples are less ideal for evolutionary research (Tosi & Coke, 2007). Moreover, sample availability has shifted from primary centers to private industry, limiting their use in basic scientific research (Morton, 2011). This use in private industry has contributed to their population decline and recent designation as an endangered species (IUCN, 2022). Our PCA confirms high genetic diversity and population subdivision within this species as compared to *M. mulatta*. Thus, the samples sequenced here can be used for future studies that necessitate an understanding of the genetic diversity of this species as an alternative to the relatively uniform populations of Mauritius.

### 4.2 Analysis of sex chromosomes confirm expected population structure

The primary signature of the Y‐chromosome PCA is the separation of allopatric *M. fascicularis* from all other populations examined. This pattern is representative of male‐mediated introgression from *M. mulatta* to *M. fascicularis* because parapatric *M. fascicularis* are in the same position as *M. mulatta* along PC1, whereas allopatric *M. fascicularis* are separated. This is in accord with previous studies in this system (Stevison & Kohn, 2009; Tosi et al., 2002). Despite accounting for over half the variance in the data, this PC was marginally significant (*p* = .049). PC2 presented an intriguing, though statistically insignificant, signature matching a pattern of character displacement. The relatively high *p*‐values, especially in contrast to those of the first two principal components of the autosomal PCA, are likely in part due to a lower sample size in both individuals and genomes. Males only make up 23 of the 38 total individuals analyzed, and the Y‐chromosome SNPs constituted roughly 1/1000th the total number of SNPs analyzed. Similarly, the X‐chromosome analysis in males and females showed the same visual patterns as each other, though significance was considerably lower in the female‐only analysis, which was represented by fewer individuals than the male‐only analysis, showing directly the effect of sample size on two datasets with essentially the same result. The X‐chromosomes do not exhibit any unusual signature of introgression, further cementing that introgression from *M. mulatta* to *M. fascicularis* is male‐driven. Instead, the X‐chromosomes largely identify the expected differentiation between species and populations within species.

### 4.3 Aggression is likely not involved in reinforcement

Based on the literature, we initially hypothesized that the increase in aggressive behavior observed in parapatric *M. fascicularis* and *M. mulatta* relative to their allopatric conspecifics may be a result of reinforcement speciation as a mechanism to prevent heterospecific mating. An integrated study of GWAS in humans, OMIM (Online Mendelian Inheritance in Man) entries, KO experiments, and transcriptomic studies in rodents resulted in 40 candidate genes for aggressive behavior in mammals (Zhang‐James et al., 2019). Thirty‐nine of these genes are annotated in the rheMac10 reference genome used for this study though none were present in our final list of 184 candidate genes for reinforcement in this system. Additionally, four of the 184 candidate genes identified by this study were identified as being involved in “Abnormal aggressive, impulsive or violent behavior” in the Human Phenotype Ontology database by gProfiler, but our literature search could not confirm this association. However, since this study did find overrepresentation of multiple neurological categories in the candidate gene list, it is possible these represent novel genes affecting aggression (see below).

### 4.4 Many genes involved in neural functioning and behavior may be involved in reinforcement

In the gProfiler analysis presented here, multiple GO and HP categories involved in neural functioning/development and behavior were overrepresented in the reinforcement candidate gene list, suggesting a large number of such genes may be involved in a reinforcement process. In addition, a single gene involved in social behavior, one of the categories subset for the literature search, NPAS4, stood out as a candidate gene. A previous study using qPCR on four genes in mice separated from their mothers (a stressor) versus a control found that out of these, only NPAS4 showed significantly upregulated expression in the prefrontal cortex in the maternally separated mice (Ryabushkina et al., 2020). A study by Coutellier et al. (2012) examined the effects of NPAS4 knockout (KO) on a variety of behaviors in mice. NPAS4 KO mice traveled a further distance in an open‐field test than wild‐type (WT) mice, indicating hyperactivity in a novel environment. In a 6‐trial social test, KO mice spent significantly less time exploring an ovariectomized female in the 1st and 2nd trials compared to WT mice and in the 1st, 2nd, and 6th trials compared to heterozygous mice. KO mice won significantly more often in tube dominance tests compared to WT than the other way around, suggesting higher dominance or aggressiveness. In a 2‐day social test, KO mice spent significantly more time avoiding a new mouse on the 1st day than WT mice did. WT and heterozygous (HET) mice had alternation levels in a Y‐maze significantly higher than chance predictions, whereas KO mice did not, indicating reduced exploratory behavior in the latter. In a place and reversal swimming test, HET mice tended to make errors or take more time. In a novel object recognition test, WT mice spent significantly more time exploring a novel object than a familiar one (presented to them 24 h beforehand), but the HET and KO mice spent equal time exploring both objects, suggesting they did not remember the previously presented object. KO mice also exhibited an increased startle response to acoustic signals, whereas HET mice were intermediate (Coutellier et al., 2012).

It is clear then that NPAS4 affects a variety of behaviors in mice, including behaviors potentially related to reinforcement such as exploration and aggression, even though the specific effects of different mutations are not fully known. Specifically, exploratory behavior as detailed above can refer to an organism's capacity to explore new environments (directly affecting capacity to migrate) and explore unfamiliar individuals (e.g., individuals from different populations). Therefore, exploratory behavior is directly connected to the capacity to hybridize and would be expected to exhibit patterns of selection in a reinforcement scenario. Nonetheless, to our knowledge, this gene has not been previously examined in macaques. The overrepresentation of multiple neurological and behavioral categories in the reinforcement candidate gene list suggests that parapatric *M. fascicularis* may have polygenic behavior associated with reinforcement, though the lack of previously known aggression genes in this dataset suggests it would not be aggressive behavior, though perhaps distinct mating preferences to prevent hybridization. Reinforcement is typically envisioned as increasing premating isolation, as this is an early stage that can act to prevent wastage of reproductive investment.

### 4.5 Several genes involved in reproduction and estrogen signaling may be involved in reinforcement

**FIGURE 5:**
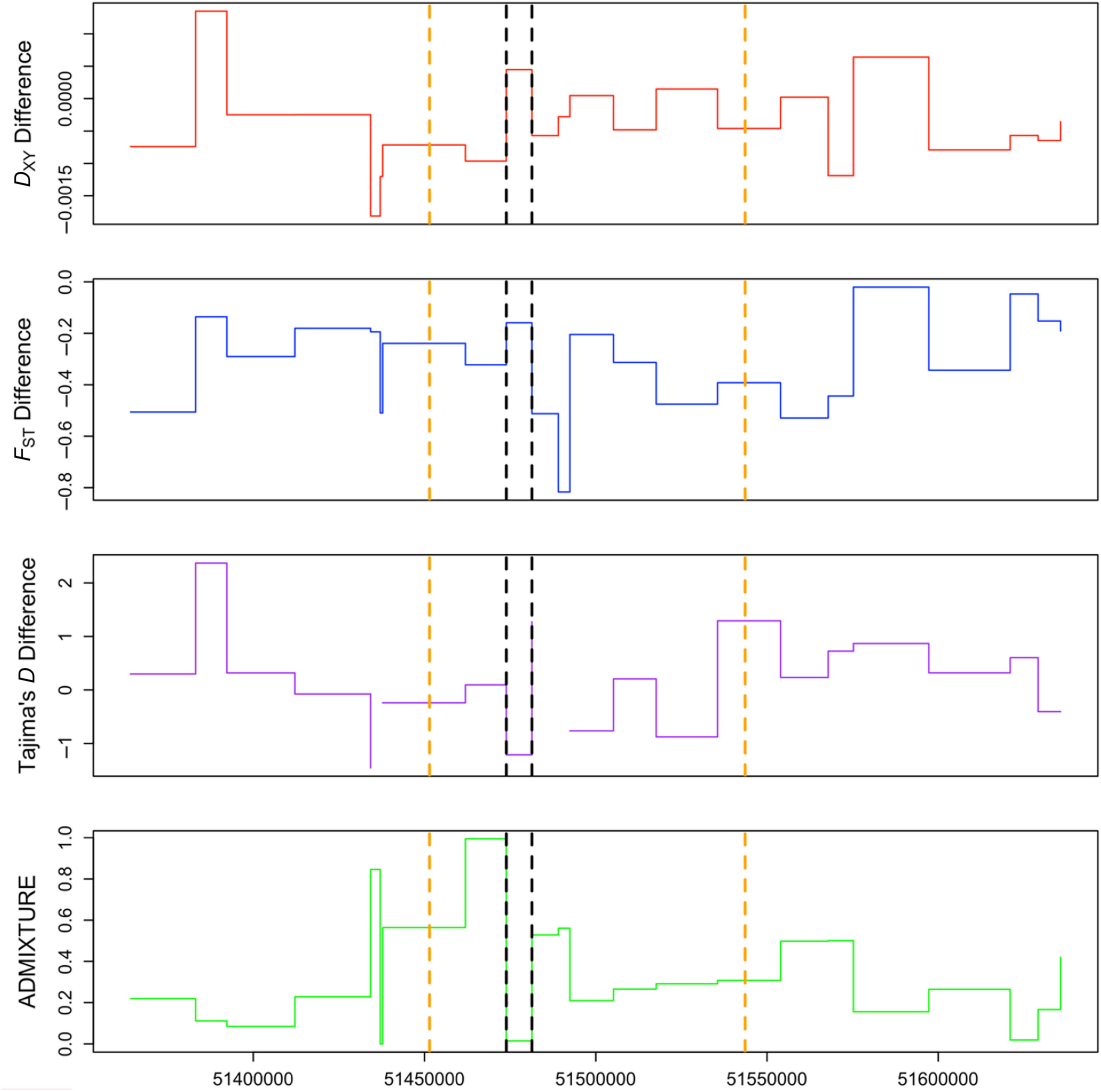
Summary statistics of the gene RARA. RARA, one of the candidate genes resulting from our analysis, is shown here as a representation of signatures in all candidate genes. From top to bottom, different panels represent *D*_XY_ Difference, *F*_st_ Difference (both defined in Equation 1), Tajima's *D* Difference (defined in Equation 2), and ADMIXTURE values. *X*‐axis represents coordinates along chromosome 16. Orange dashed lines signify total length of gene as annotated by Ensembl. Black dashed lines signify the window of the gene that was considered a significant candidate window in our analysis. This window has an unusually high *D*_XY_ Difference and *F*_st_ Difference as well as an unusually low Tajima's *D* Difference and ADMIXTURE, whereas other windows in this figure may fulfill one or more of these conditions but not all simultaneously.

Multiple reproductive genes have genomic windows overlapping them that show signatures of genomic character displacement. The reproduction candidate genes (RARA, M1AP, TRIP13, RNF216, AMHR2, and CLP1) in our analysis are known to be crucial to a variety of reproductive functions. The binding of RARA (Retinoic Acid Receptor‐α) to retinoic acid is necessary for the generation of Müllerian ducts (a developmental precursor to uterine tubes) and the maintenance of epithelia in adult female reproductive tracts (Hewitt et al., 2020). RARA itself is activated by ERα (Estrogen Receptor‐α), and the specific binding site has been confirmed to fall within the first intron of RARA in humans using chromatin immunoprecipitation (ChIP) cloning of human breast cancer cells (Laganière et al., 2005). Additionally, ChIP‐seq in mice confirmed the presence of a binding site with great affinity for ERα (termed a super‐enhancer) over a large region of RARA including upstream of the genes as well as the first and second introns of most of the transcripts (Hewitt et al., 2020). Notably, our candidate genomic window for reinforcement selection falls within the first intron of two RARA transcripts (Figure 5). Using motif analysis in this study, it was found that within this window, there are 39 transcription factors predicted to bind to the allele unique to the parapatric *M. fascicularis* population. Narrowing down to only alleles that are fixed within the population as well, there are four predicted transcription factors. In addition to this, a gene whose expression is known to be greatly upregulated in the presence of estradiol, ADCY2, was a candidate gene in the current study. This regulation is mediated by interactions between estradiol and miRNA's that bind to the 3′UTR of ADCY2 (Zhao et al., 2020). The motif analysis presented here found that there are 40 transcription factors predicted to bind to the unique parapatric *M. fascicularis* allele in the 3′UTR of this gene.

AMHR2 (Anti‐Müllerian Hormone Receptor 2) is a crucial gene for the deterioration of Müllerian ducts in developing males. Mutations in this gene in humans are the primary cause of a disorder where men develop uteri (Josso & Picard, 2022). This developmental pathway is highly conserved in vertebrates (Adolfi et al., 2019), so it is likely involved in a similar process in macaques.

M1AP (Meiosis‐1 Associated Protein) is a crucial gene for spermatogenesis in humans and mice. Genotyping and whole‐exome studies in humans have identified mutations in this gene as a cause of oligospermia (low sperm count) and azoospermia (no sperm produced due to meiotic arrest) in people from China, Croatia, Germany, Poland, Portugal, the Netherlands, Turkey, and the UK (Tu et al., 2020; Wyrwoll et al., 2020). Additionally, expression of this gene in mice is strongest in early spermatogenesis, and knockout (KO) experiments result in meiotic arrest in males with no such effect in females (Arango et al., 2013).

TRIP13 (Thyroid Hormone Receptor Interactor 13) is necessary for double‐strand break repair in mice, which has been demonstrated by periodic acid‐Schiff and terminal deoxynucleotidyl transferase dUTP nick end labeling staining for DNA fragmentation in spermatocytes and oocytes of wild‐type (WT) and mutant TRIP13 mice, where the latter have improper double‐strand break repair and gametes are not generated properly (Roig et al., 2010).

RNF216 (Ring Finger Protein 216) is necessary for testis development in mice and gonadogenesis in men and women. KO mice (using lac operon‐replacing exons) were generated that had significantly smaller testis than WT or heterozygous mice and were completely sterile. Female KO mice were not significantly more sterile than WT mice (Melnick et al., 2019). Similar KO experiments using CRISPR‐Cas9 gave the same results, though double‐staining spermatocytes with antibodies against SYCP1 and SYCP3 identified specifically that spermatogenic arrest occurred at the zygotene stage of meiosis I (Li et al., 2021, p. 216). Whole‐exome sequencing of three siblings in a Palestinian family exhibiting ataxia (neurological disfunction affecting movement) and hypogonadotropic hypogonadism (hormonal disorder in the hypothalamus/pituitary gland causing abnormal puberty and gonadogenesis in both men and women) found 13 homozygous variants predicted to be rare and deleterious in the third patient, where only two were shared between all three siblings (a mutation each in RNF216 and OTUD4). These variants were not present in unaffected family members. These two genes were then sequenced in nine affected unrelated people from seven unrelated families, and various mutations were found in RNF216 in four individuals (Margolin et al., 2013).

The homolog of CLP1 (cbc) in *Drosophila* has been demonstrated to be necessary for spermatogenesis, and the function of this gene can be recovered in KO flies using human and mouse orthologs, suggesting a similar function (Wu et al., 2021).

Clearly, then, mutations in these genes (in mice and humans) result in disorders of sex development and improper gametogenesis. These are also signatures of reproductive incompatibilities in hybrids and may account for the observed reduced fecundity of parapatric *M. fascicularis* (Kumpai et al., 2022). It could be speculated that this is a negative selection pressure that reinforcement speciation acts to prevent. However, given that the genes here exhibit signatures of divergent selection (decreased Tajima's *D* in parapatric *M. fascicularis* and increased *F*_ST_/*D*_XY_ between parapatric populations) that we searched for in this study, their presence in our candidate gene list suggests they are themselves selected for by reinforcement. As mentioned before, reinforcement is typically conceived as a process that selects for prezygotic barriers when postzygotic isolation is already present (Coyne & Orr, 2004; Dobzhansky, 1937). However, a process analogous to this concept of reinforcement speciation was originally proposed where postzygotic barriers themselves may be reinforced (Wallace, 1889). Although Dobzhansky brought the insight that reinforcement will more typically act on prezygotic barriers, he did not exclude the possibility of it acting on postzygotic barriers (Dobzhansky, 1937). Additionally, W. D. Hamilton suggested that the stage of reproductive isolation at which reinforcement acts should depend on relative costs and benefits, not an arbitrary line at zygote formation (Butlin & Smadja, 2018; Cronin, 1991). This thinking seems to fall more in line with a view of reproductive isolation (and speciation as a whole) as a continuum (Westram et al., 2022). Therefore, the exact stage at which these genes operate in this system is unclear for now (e.g., if they are involved the prezygotic or postzygotic isolation), though it is worth further investigation.

As mentioned above, one of our candidate windows falls within RARA, an estrogen‐binding gene critical for female reproductive tract development, among other functions (Hewitt et al., 2020). This window specifically falls in a region that has been demonstrated to be the primary enhancer for estrogen‐binding in human and mouse cells. Our motif analysis shows that an allele fixed within the parapatric *M. fascicularis* samples and absent in all other samples examined here has four unique predicted transcription factors compared to the reference allele. This suggests RARA transcription is differentiated in parapatric *M. fascicularis* from all other population groupings. Additionally, the motif analysis in the present study identified 40 transcription factors that uniquely bind to alternate alleles unique to parapatric *M. fascicularis* for the 3′UTR of ADCY2, a known interactor with estrogen. However, these alternate alleles were not fixed in parapatric *M. fascicularis*, and our candidate window did not fall in this region of the gene. In macaques, the estrogen signaling pathway is critical for red sexual swelling (Dixson, 1998; Rhodes et al., 1997; Vandenbergh, 1965), a phenotype that differentiates *M. mulatta* and *M. fascicularis*. The former exhibits red sexual swelling of the face down to the thighs, whereas the latter has swelling only above the thighs (Fooden, 2006). Both male and female *M. mulatta* sexually select for this phenotype in the other sex (Dubuc, Allen, et al., 2014; Higham et al., 2021), and this trait is genetically heritable. An analysis combining male and female *M. mulatta* computed the narrow‐sense heritability of facial skin redness and darkness, respectively, as 0.118 and 0.125 (Dubuc, Winters, et al., 2014). First, this suggests that these phenotypes are genetically heritable, which is a precondition for selection to act on them. Second, it suggests that *M. mulatta* has the capacity for assortative mating, which is typically considered a precursor to reinforcement (Servedio, 2000). Nonetheless, as stated in the introduction, the effectiveness of assortative mating in leading to reinforcement can vary depending on the direction of the introgression. Indeed, in a scenario with one population solely introgressing into the other, it is expected that a preference for a specific trait within a population is more likely to lead to reinforcement (Servedio, 2000). Thus, it is necessary to study the basis of sexual selection in *M. fascicularis*, which is not well understood, though some evidence suggests direct female choice for mates plays a small role in male success (Engelhardt et al., 2006). Nonetheless, *M. fascicularis* does exhibit sexual swelling, which is generally a sexually selected trait in primates (Dixson, 1998). If parapatric *M. fascicularis* also sexually select on the basis of sexual swelling, genes like RARA and ADCY2 may be involved in a reinforcement phenotype, though further studies will be needed to confirm this.

## 5 CONCLUSIONS

Our analysis finds 184 (less than 1% of the genome) genes exhibiting a signature of genomic reproductive character displacement. The overrepresentation of various neurological categories and the presence of eight estrogen signaling and reproductive genes in our candidate gene dataset suggest a polygenic process affecting multiple reproductive barriers. Our study suggests that reinforcement may have contributed to the divergence of these species by acting through multiple biological processes such as behavioral mating preferences, sexual swelling, and reproductive dysfunction in hybrid crosses. Future studies should examine the functional impacts of variants in these genes in the lab or in nature to assess the validity of these candidate genes. Similarly, future studies should be conducted to examine the extent of reproductive isolation presently caused by mating preferences, mating cues, and intrinsic reproductive dysfunction to further elaborate on possible mechanisms of reinforcement.

## AUTHOR CONTRIBUTIONS

**Nick Bailey:** Conceptualization (supporting); data curation (equal); formal analysis (lead); methodology (lead); software (lead); visualization (lead); writing – original draft (lead); writing – review and editing (equal). **Cody Ruiz:** Conceptualization (supporting); data curation (equal); methodology (supporting); resources (equal); visualization (supporting); writing – original draft (supporting); writing – review and editing (equal). **Anthony Tosi:** Conceptualization (supporting); data curation (equal); resources (equal); writing – original draft (supporting); writing – review and editing (equal). **Laurie Stevison:** Conceptualization (lead); data curation (equal); funding acquisition (lead); project administration (lead); resources (equal); writing – original draft (supporting); writing – review and editing (equal).

### OPEN RESEARCH BADGES

This article has earned an Open Data badge for making publicly available the digitally‐shareable data necessary to reproduce the reported results. The data is available at https://github.com/StevisonLab/Reinforcement‐Project.

## Supporting information


Figure S1.

Figure S2.

Table S1.

Table S2.

Table S3.

Data S1.

## Data Availability

All custom scripts, MultiQC summary data, and SnpEff summary data for in this analysis can be found on the following page: https://github.com/StevisonLab/Reinforcement‐Project. gProfiler results can be found on the following page: https://biit.cs.ut.ee/gplink/l/3CcJdHqYSa. A UCSC custom track with all windowed population genetic statistics can be found at https://genome.ucsc.edu/s/npb596/Reinforcement_Data. FASTQ files uploaded to NCBI SRA under BioProject PRJNA976321.
