# Supplementary figures and images for: Genomic analysis of the rhesus macaque (Macaca mulatta) and the cynomolgus macaque (Macaca fascicularis) uncover polygenic signatures of reinforcement speciation
Source: Ecol Evol. 2023 Oct 15;13(10):e10571. doi: 10.1002/ece3.10571 (PMC10577069; doi:10.1002/ece3.10571)

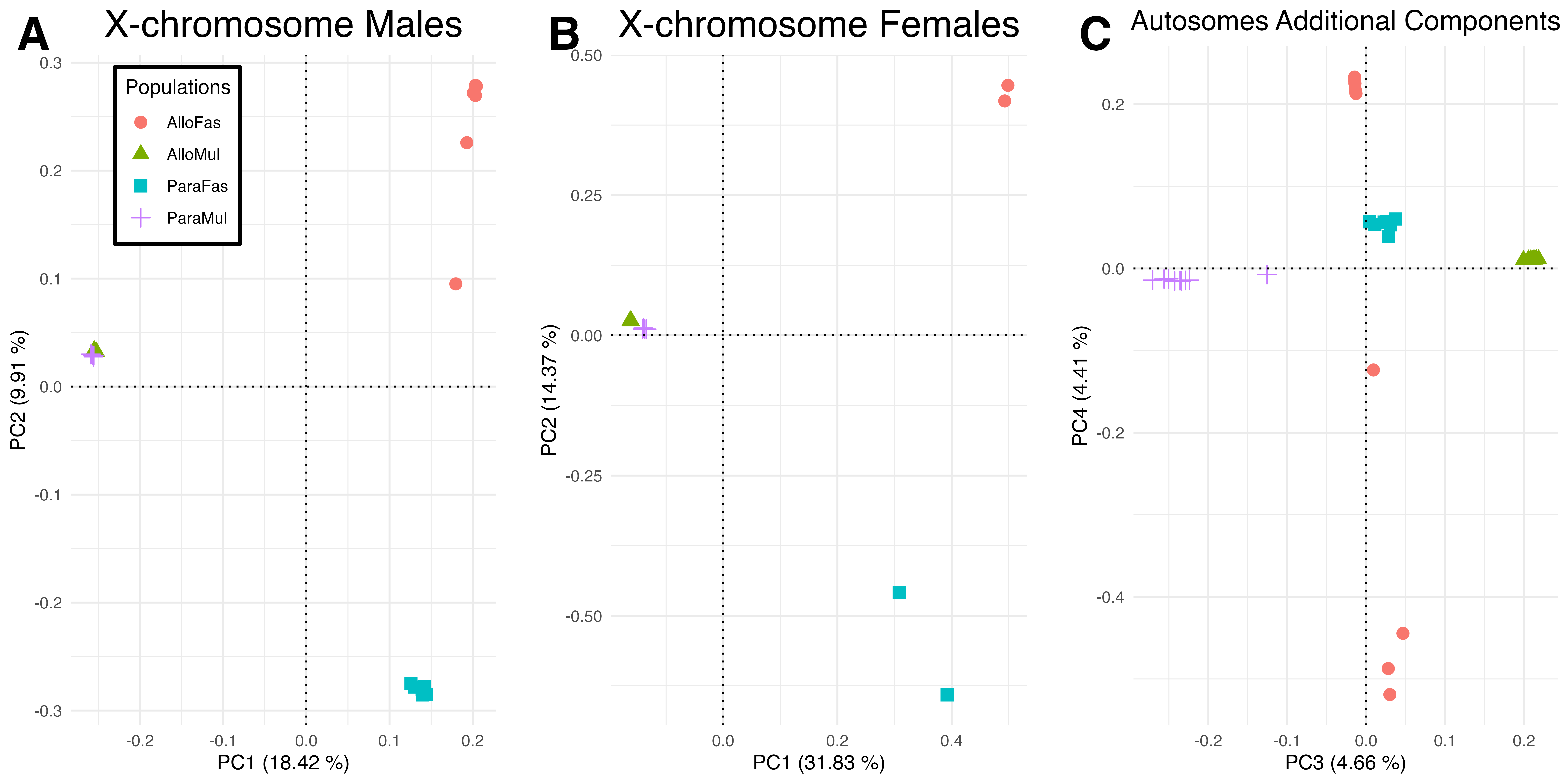

Supplement: Supplementary file 1 — Figure S1. [file ECE3-13-e10571-s006.png]
