# Supplementary material for: Genomic analysis of the rhesus macaque (Macaca mulatta) and the cynomolgus macaque (Macaca fascicularis) uncover polygenic signatures of reinforcement speciation
Source: Ecol Evol. 2023 Oct 15;13(10):e10571. doi: 10.1002/ece3.10571 (PMC10577069; doi:10.1002/ece3.10571)
Supplement: Supplementary file 2 — Figure S2. [file ECE3-13-e10571-s002.pdf]

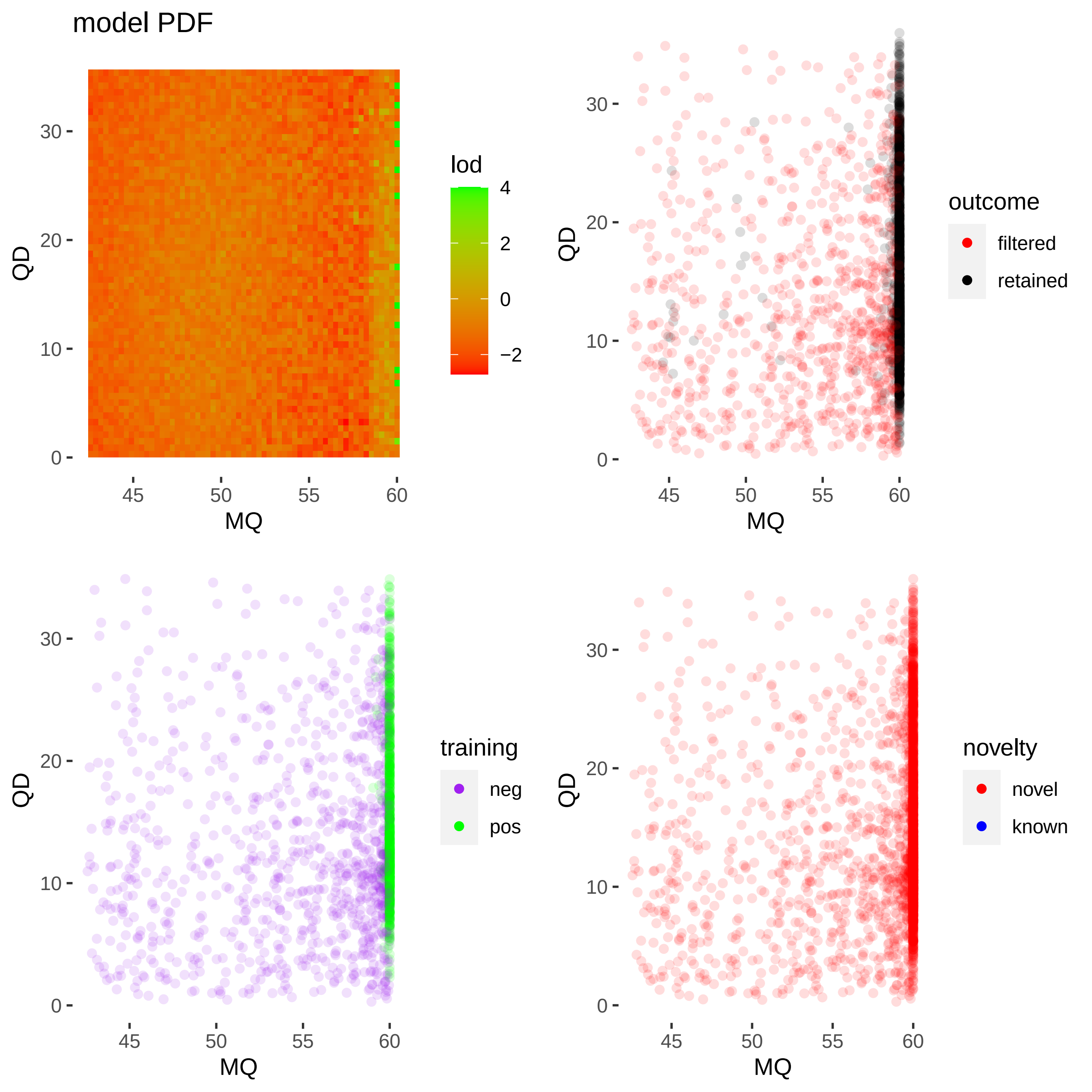
model PDF
lod
4
2
0
−2
QD
MQ
30
20
10
0
45
50
55
60
outcome
filtered
retained
training
neg
pos
novelty
novel
known

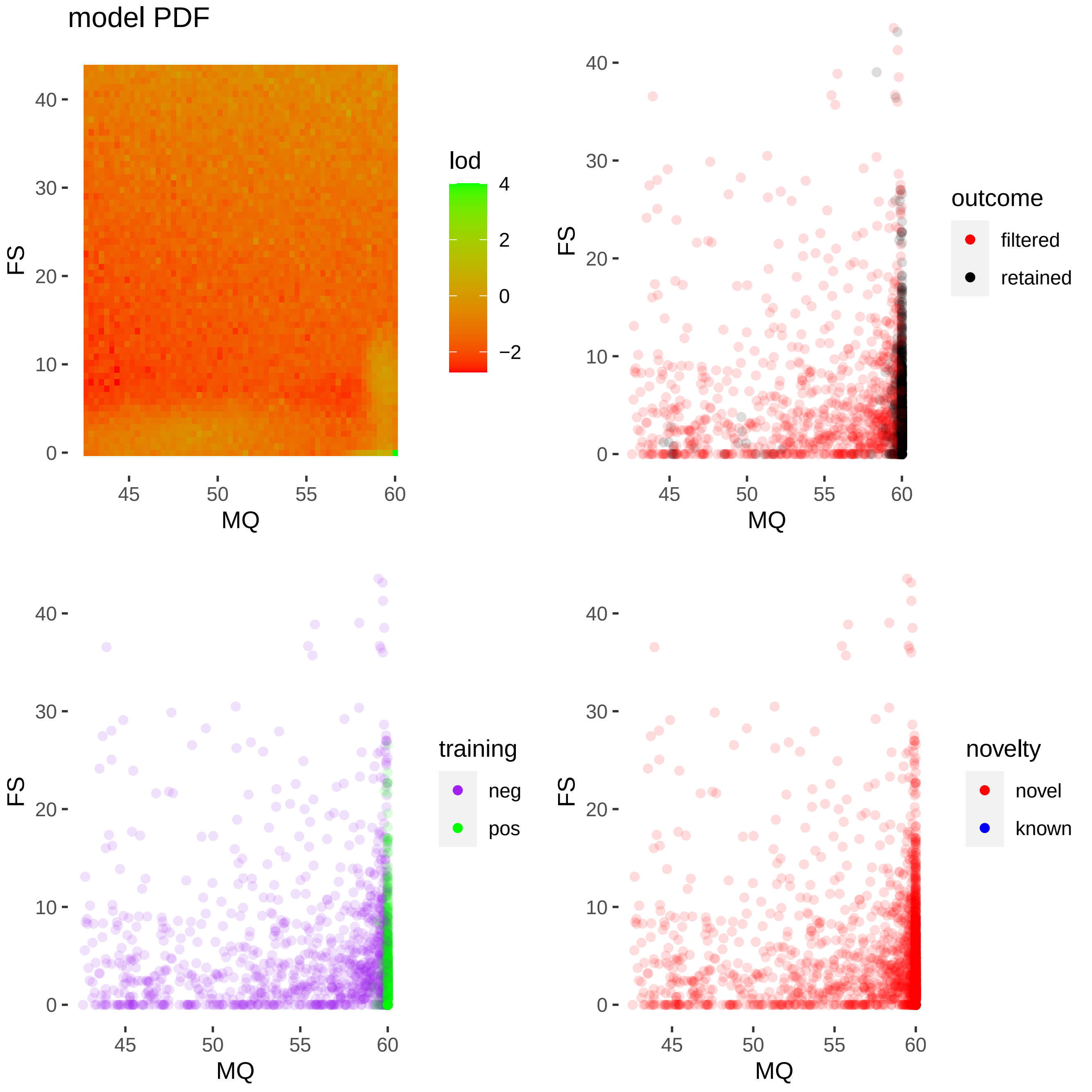

model PDF
lod
4
2
0
−2
FS
MQ
outcome
filtered
retained
training
neg
pos
novelty
novel
known

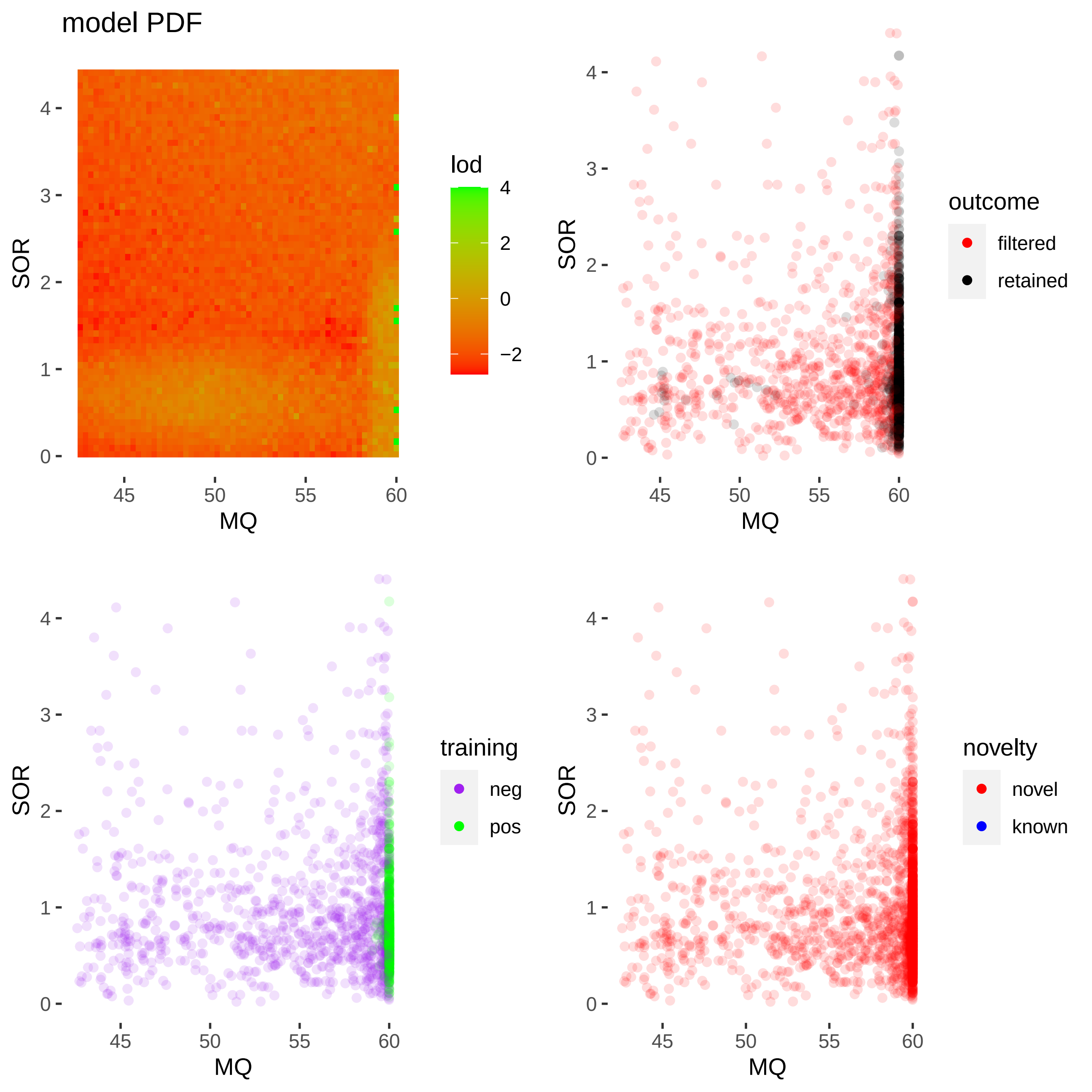
model PDF
lod
4
2
0
−2
SOR
MQ
outcome
filtered
retained
training
neg
pos
novelty
novel
known

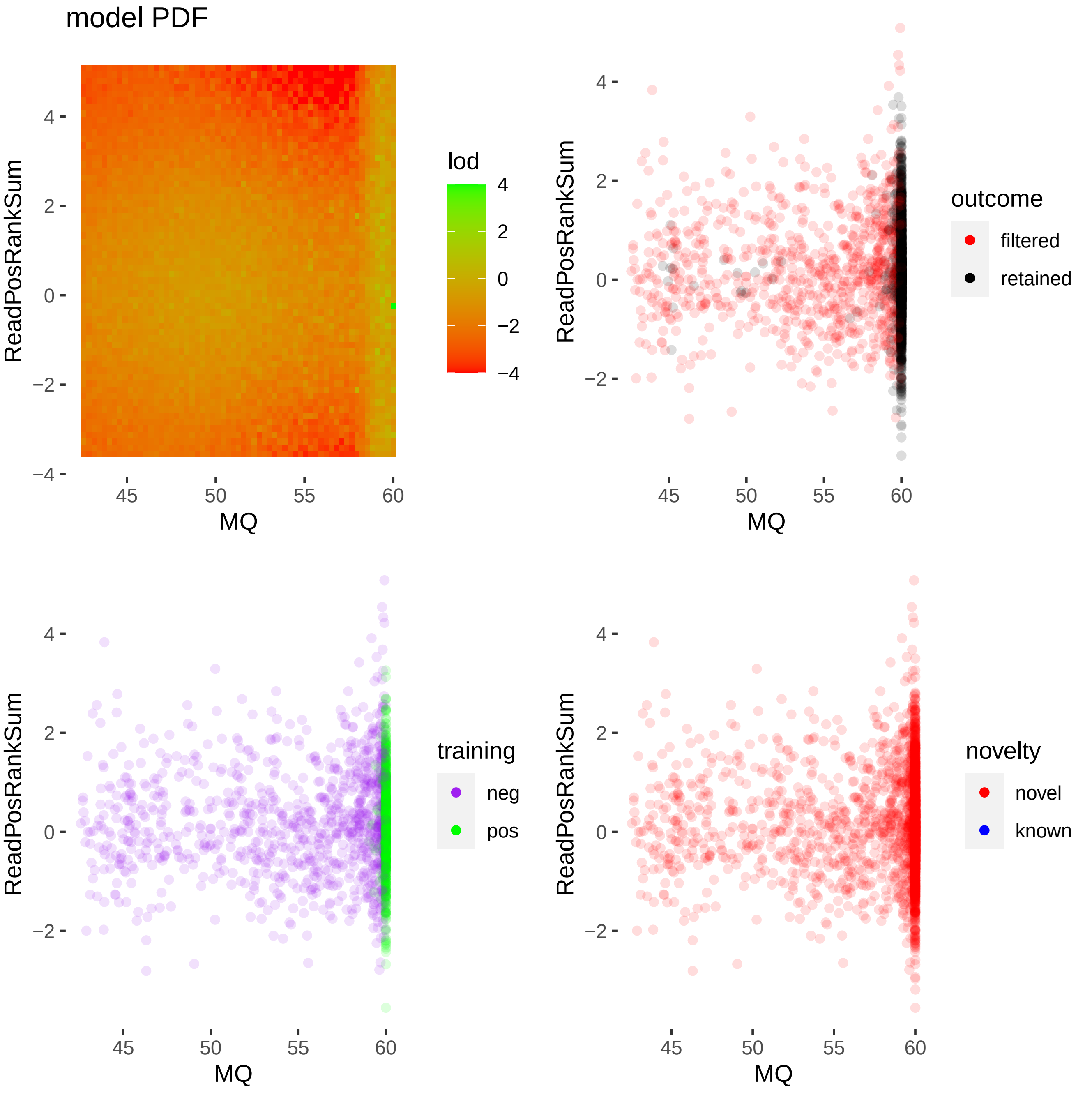
model PDF
ReadPosRankSum
MQ
lod
4
2
0
−2
−4
45
50
55
60
outcome
filtered
retained
training
neg
pos
novelty
novel
known

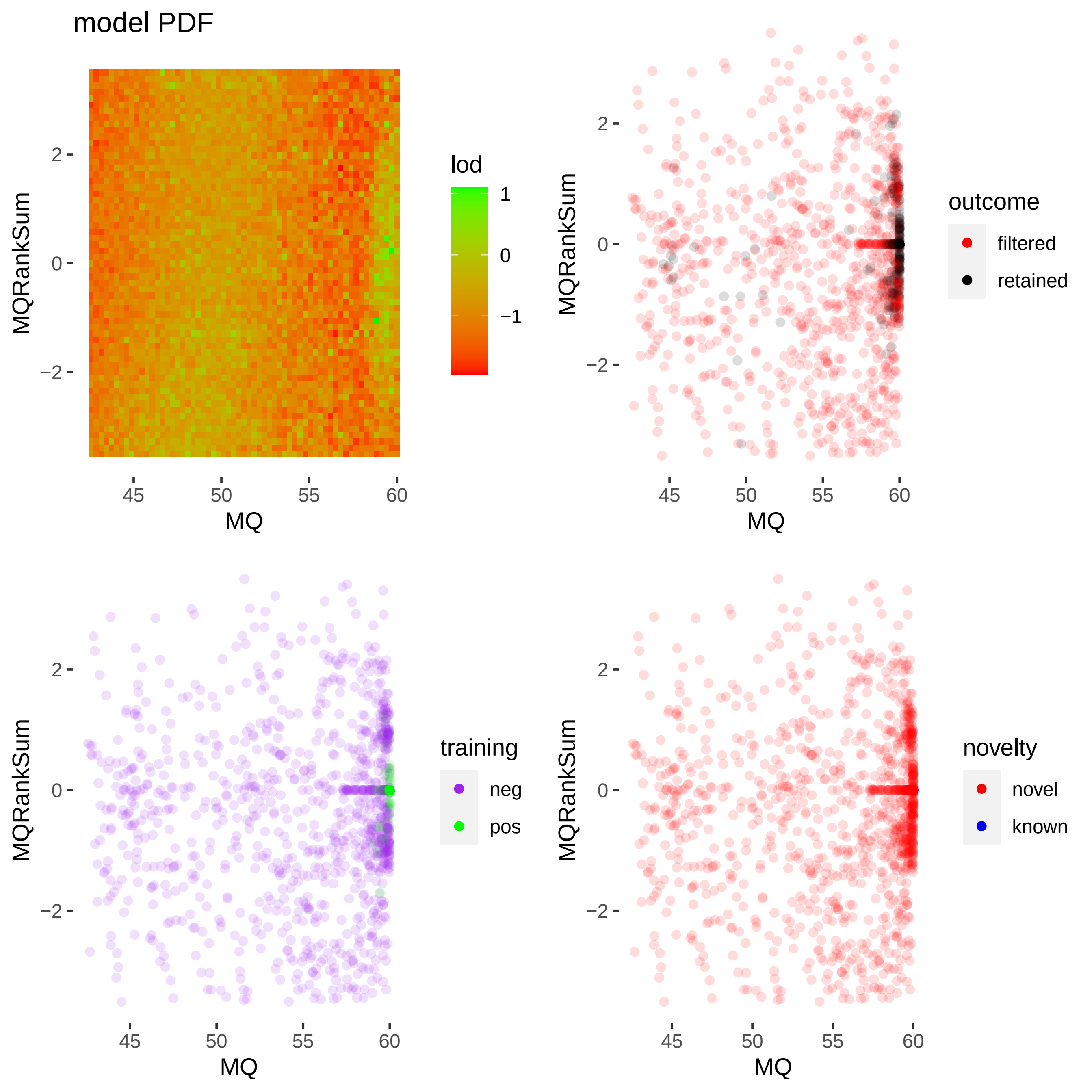
model PDF
lod
1
0
−1
MQRankSum
MQ
45
50
55
60
2
0
−2
outcome
filtered
retained
training
neg
pos
novelty
novel
known

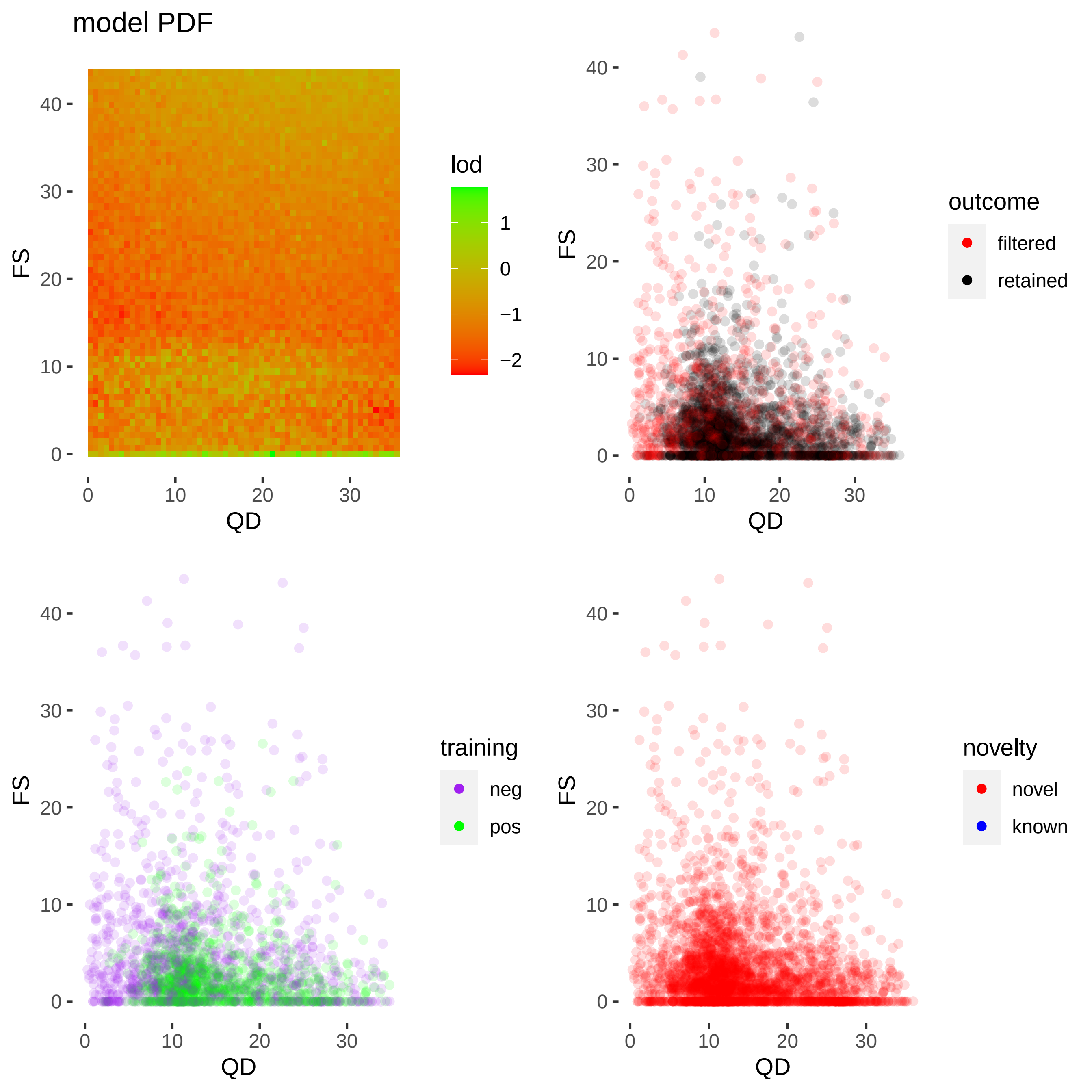
model PDF
lod
1
0
−1
−2
FS
QD
0
10
20
30
40
outcome
filtered
retained
training
neg
pos
novelty
novel
known

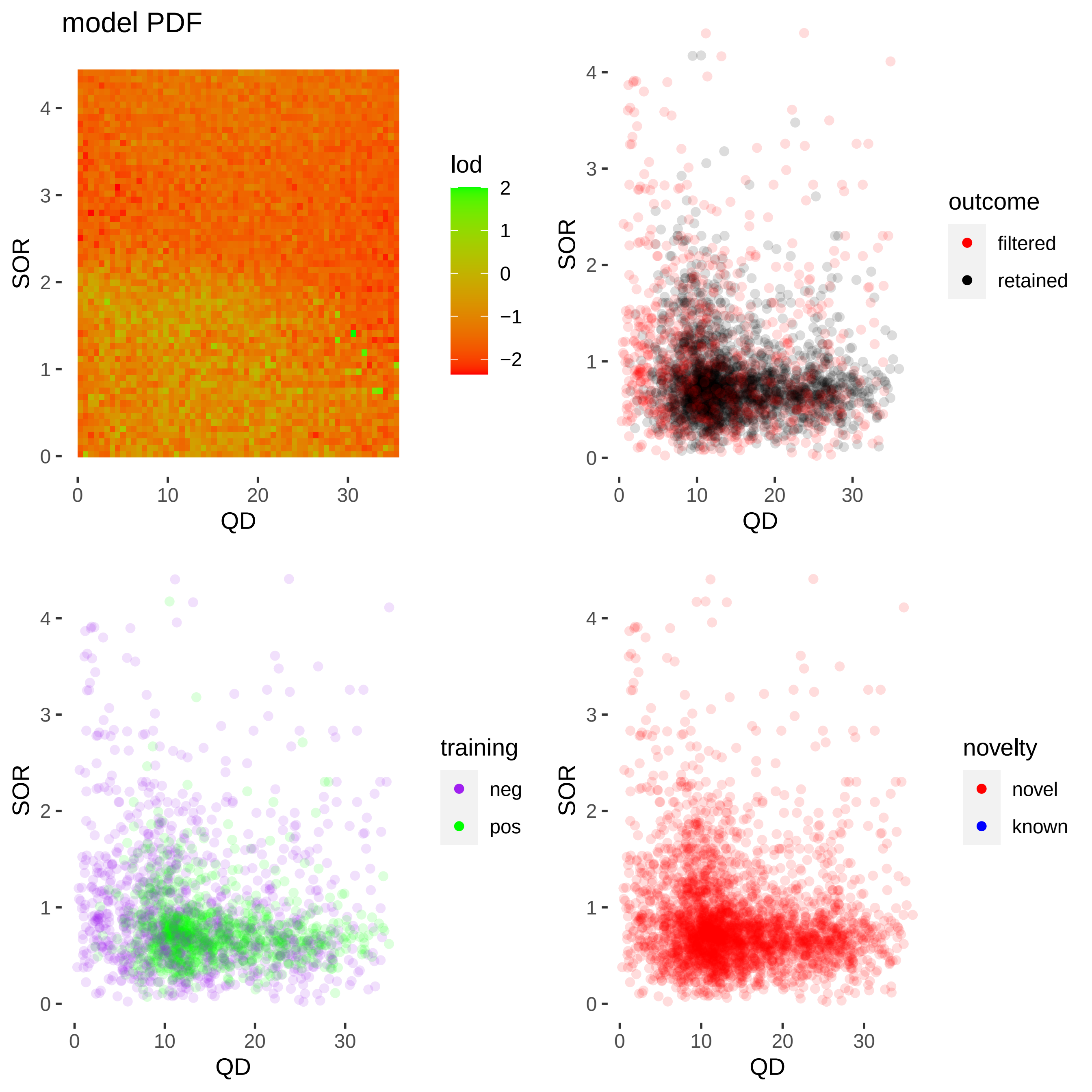
model PDF
lod
2
1
0
−1
−2
SOR
QD
outcome
filtered
retained
training
neg
pos
novelty
novel
known
0
1
2
3
4
0
10
20
30

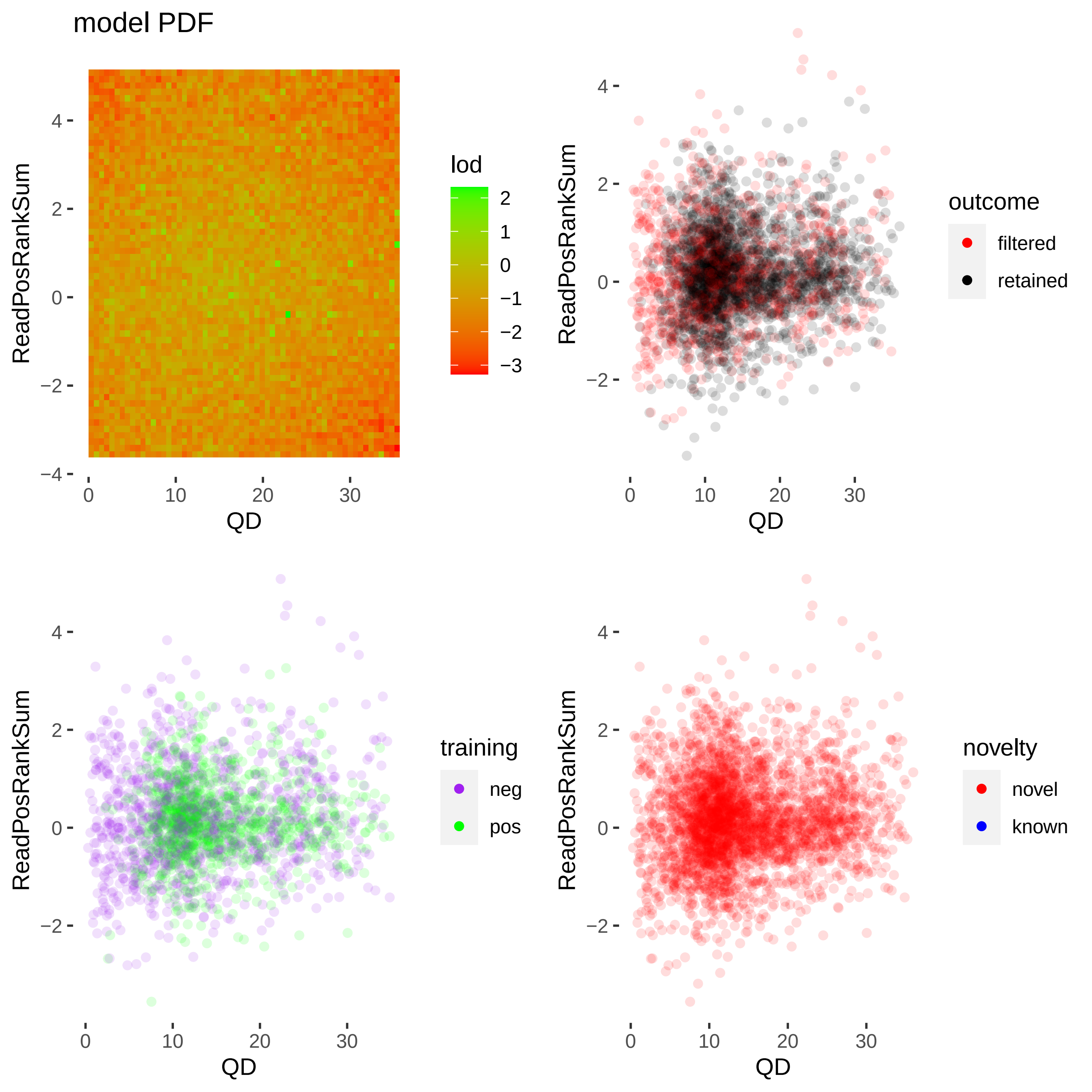

model PDF
lod
ReadPosRankSum
QD
outcome
filtered
retained
training
neg
pos
novelty
novel
known

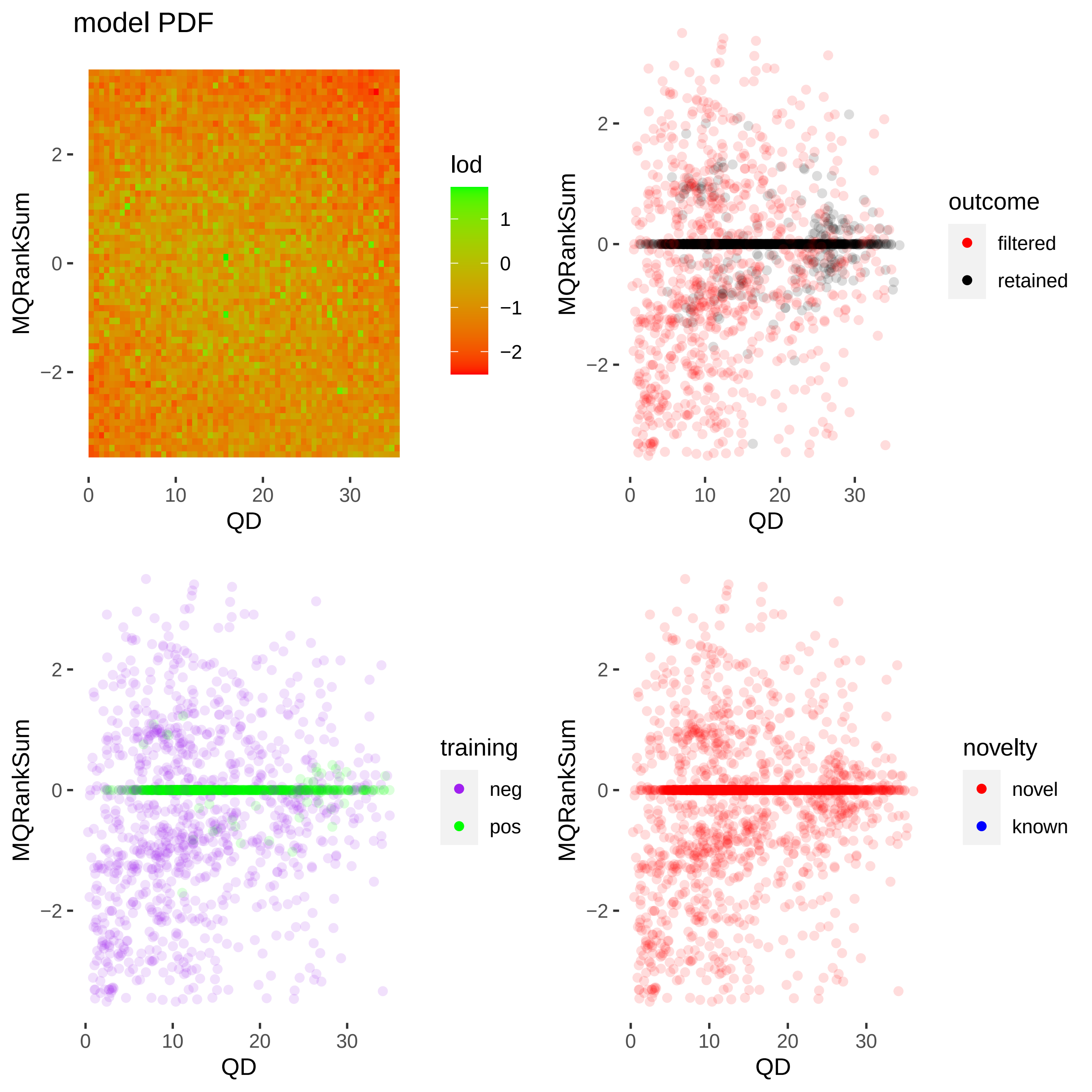

model PDF
lod
1
0
−1
−2
MQRankSum
QD
outcome
filtered
retained
training
neg
pos
novelty
novel
known

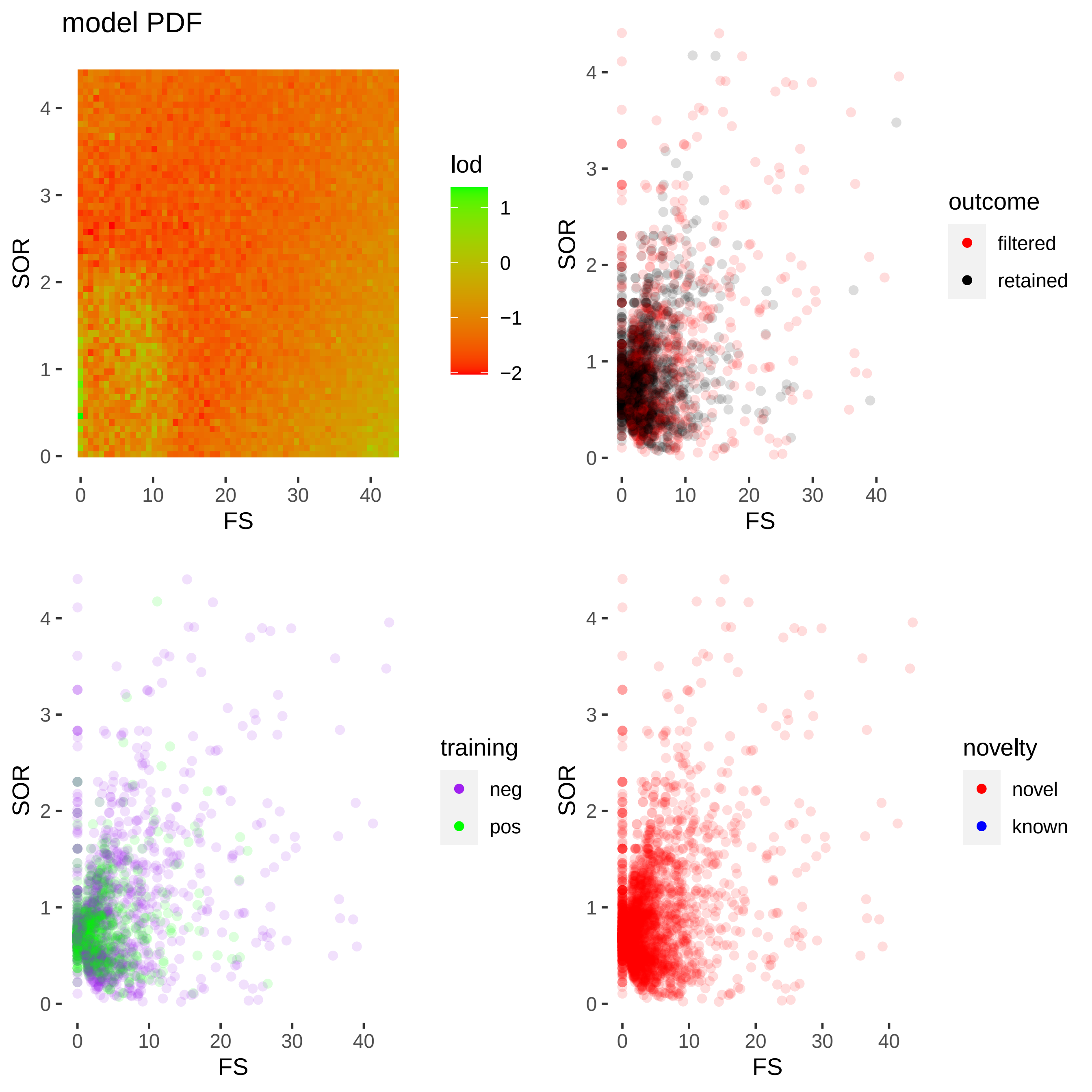
model PDF
lod
1
0
−1
−2
SOR
FS
outcome
filtered
retained
training
neg
pos
novelty
novel
known
0
1
2
3
4
0
10
20
30
40

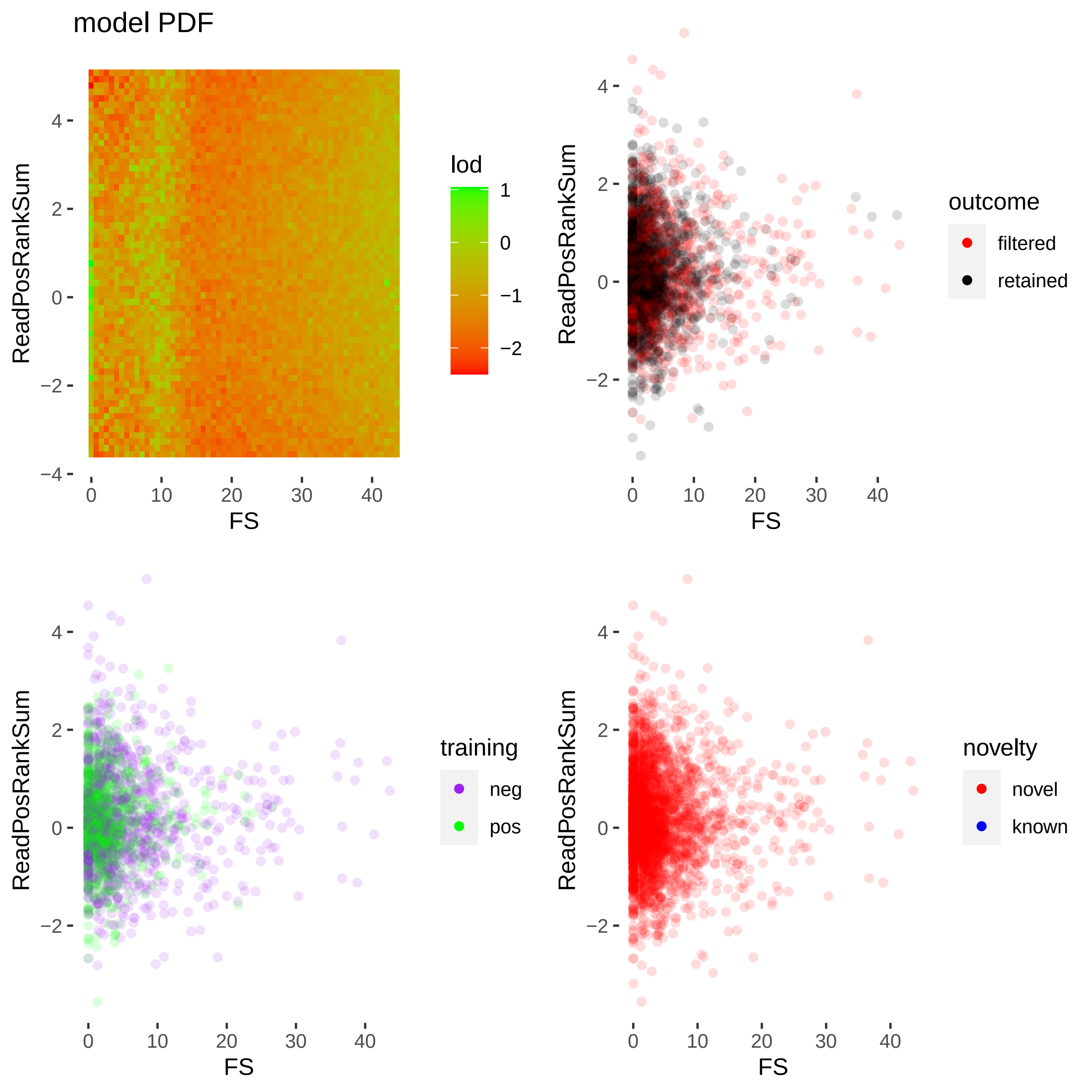

model PDF
ReadPosRankSum
FS
lod
outcome
filtered
retained
training
neg
pos
novelty
novel
known

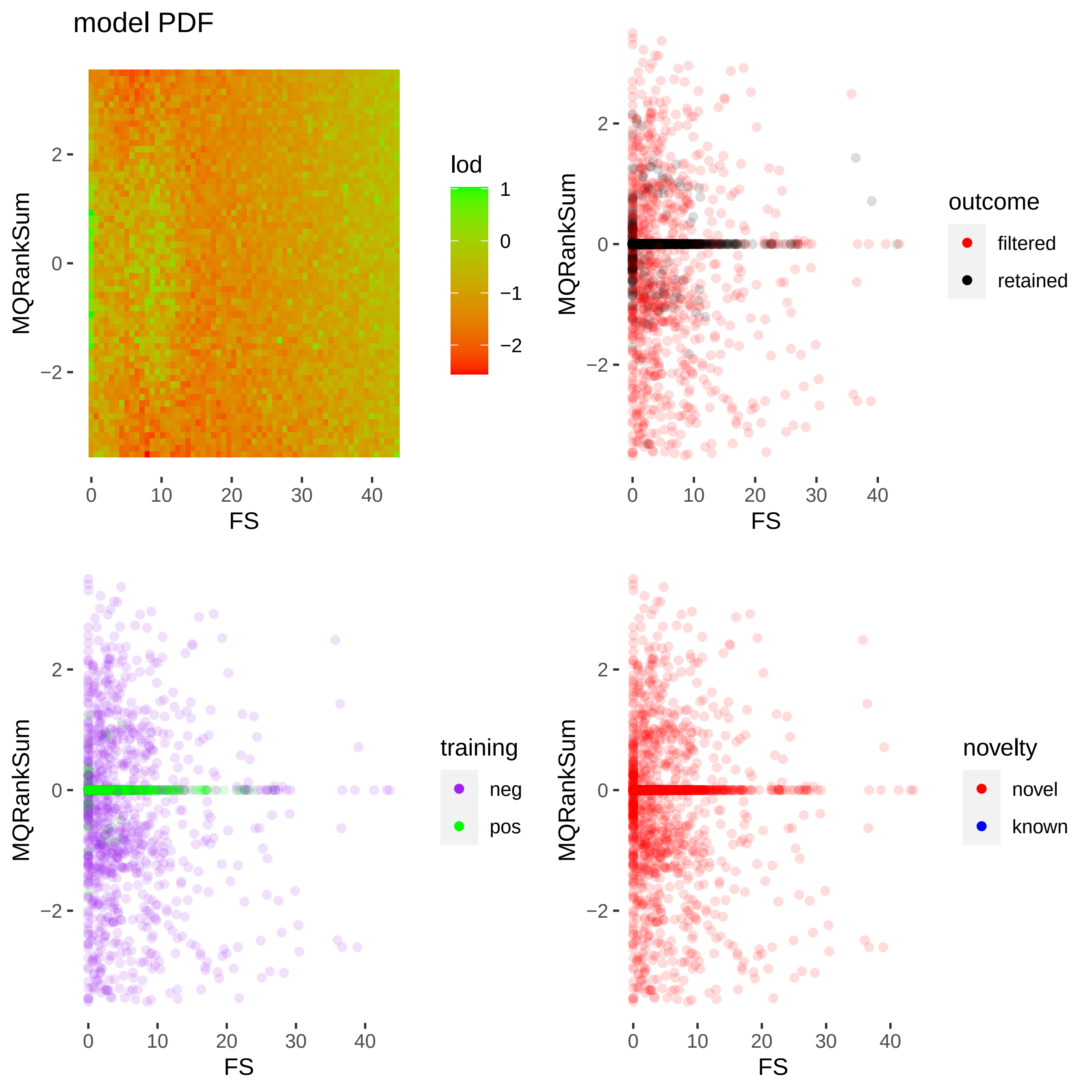

model PDF
lod
1
0
−1
−2
MQRankSum
FS
outcome
filtered
retained
training
neg
pos
novelty
novel
known

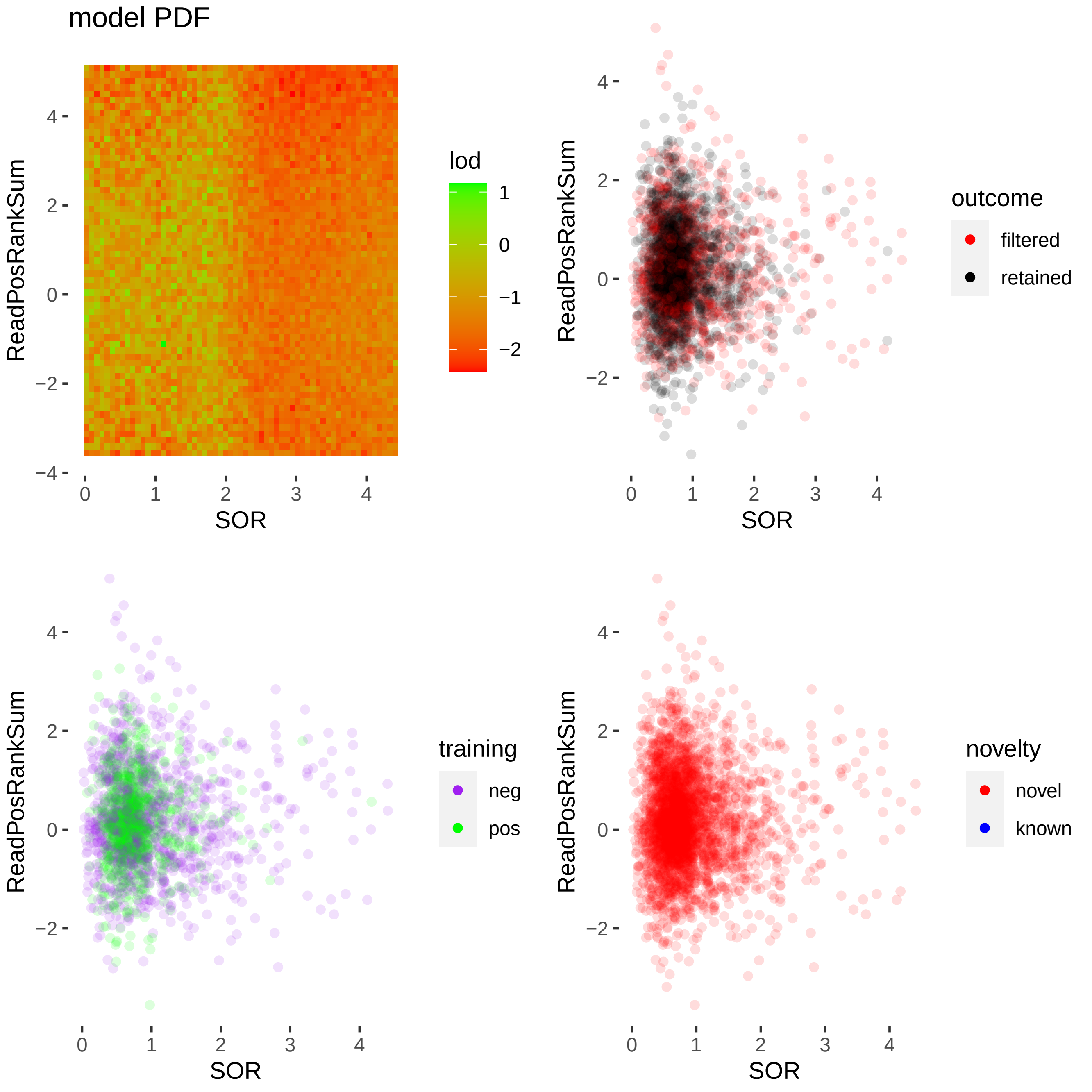

model PDF
ReadPosRankSum
SOR
lod
1
0
−1
−2
4
2
0
−2
−4
0
1
2
3
4
outcome
filtered
retained
training
neg
pos
novelty
novel
known

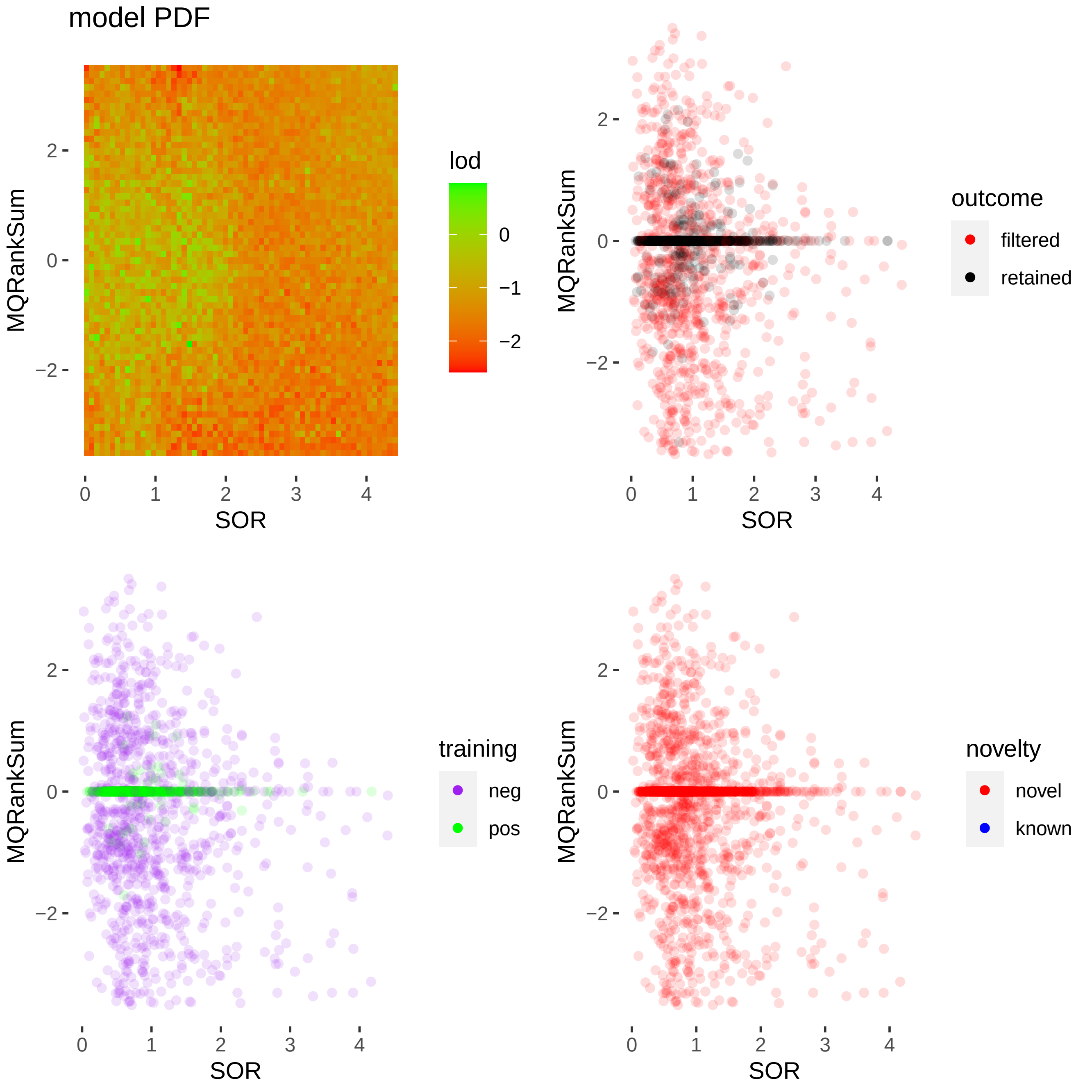

model PDF
lod
0
−1
−2
MQRankSum
SOR
outcome
filtered
retained
training
neg
pos
novelty
novel
known

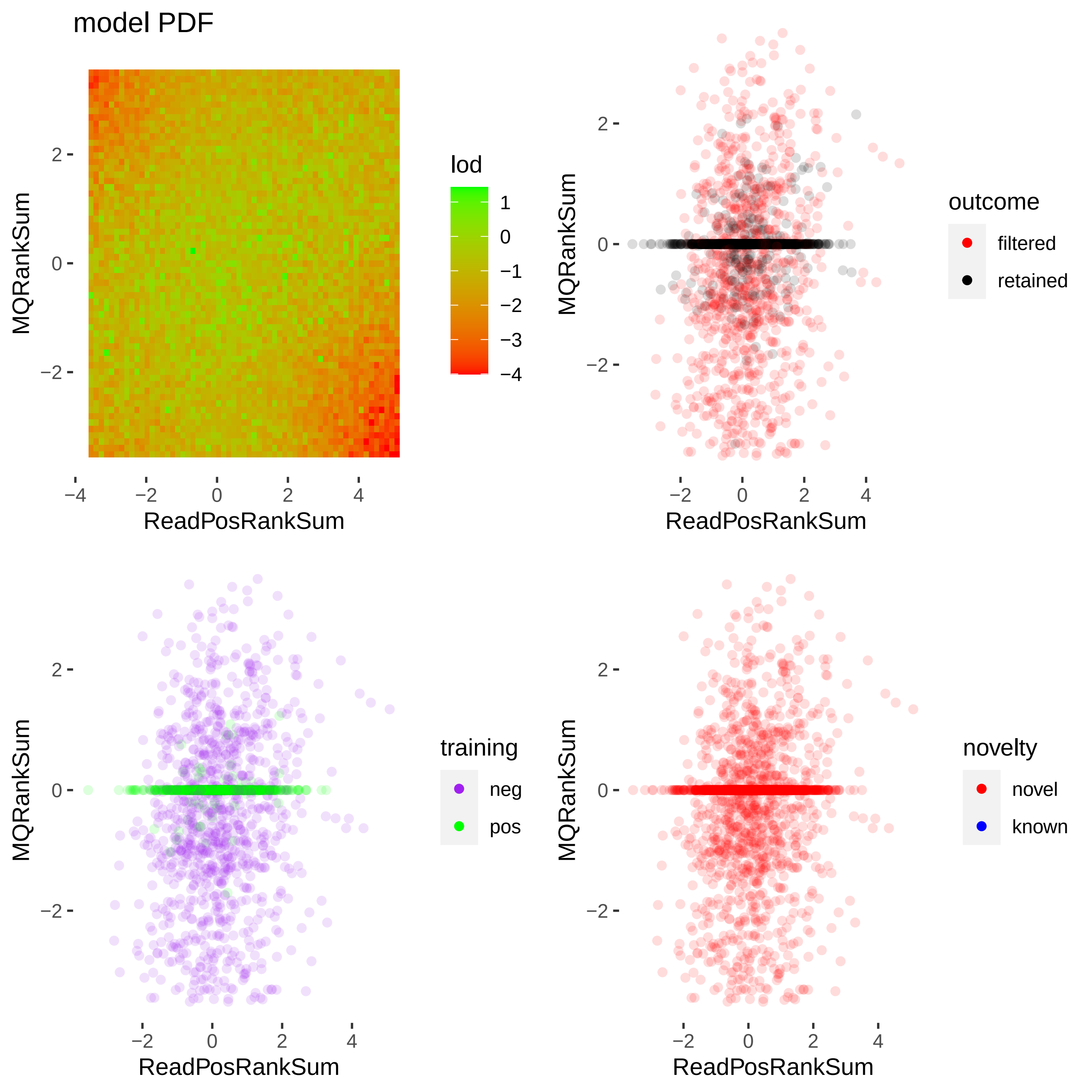
model PDF
lod
outcome
filtered
retained
training
neg
pos
novelty
novel
known
MQRankSum
ReadPosRankSum
