# Supplementary material for: Genomic analysis of the rhesus macaque (Macaca mulatta) and the cynomolgus macaque (Macaca fascicularis) uncover polygenic signatures of reinforcement speciation
Source: Ecol Evol. 2023 Oct 15;13(10):e10571. doi: 10.1002/ece3.10571 (PMC10577069; doi:10.1002/ece3.10571)
Supplement: Supplementary file 6 — Data S1. [file ECE3-13-e10571-s005.docx]

Here is the requested information.

Figure S1 Caption

Figure S1. PCA plots for additional chromosomes and autosomal components. Males and femals separated in X-chromosome analysis due to different variances. A) Plot for X-chromosome in male samples only. B) Plot for X-chromosomes in females only. C) Plot of principal components 3 and 4 for autosomes (corresponding to Figure 1A). Population labeling for all plots is as follows: AlloFas = Allopatric M. fascicularis, AlloMul = Allopatric M. mulatta, ParaFas = Parapatric M. fascicularis, ParaMul = Parapatric M. mulatta.

Figure S2 Caption

Figure S2. Plots of Variant Quality Score Recalibration metrics for samples in this study. All pairwise comparisons of the following metrics are shown in the plots: MQ (root mean square Mapping Quality), QD (Quality by Depth), FS (Fisher Strand bias), SOR (Strand Odds Ratio), ReadPosRankSum (Read Position Rank Sum), and MQRankSum (Mapping Quality Rank Sum test). More detail on the meaning of these metrics is provided in the following GATK documentation: [https://gatk.broadinstitute.org/hc/en-us/articles/360035890471-Hard-filtering-germline-short-variants](https://urldefense.com/v3/__https:/gatk.broadinstitute.org/hc/en-us/articles/360035890471-Hard-filtering-germline-short-variants__;!!N11eV2iwtfs!qPOHtENdlKSByU6qRfTKO987rMHGti7ePZwBWykMyKuhISZwSWpvnY40CHhcN1mNLCAxnZ935YI$). All plots generated by GATK VariantRecalibrator and details on the VQSR procedure and interpretation of plots can be found here: [https://gatk.broadinstitute.org/hc/en-us/articles/360035531612-Variant-Quality-Score-Recalibration-VQSR-](https://urldefense.com/v3/__https:/gatk.broadinstitute.org/hc/en-us/articles/360035531612-Variant-Quality-Score-Recalibration-VQSR-__;!!N11eV2iwtfs!qPOHtENdlKSByU6qRfTKO987rMHGti7ePZwBWykMyKuhISZwSWpvnY40CHhcN1mNLCAx6_AgyKg$).

Table S1 Caption

Table S1. Extended data for samples used in this study. Sheet 1 contains all data from Table 1 and additional data on sequencing and read information. Sheet 2 contains detailed read information and values used for calculations of initial read coverage in Sheet 1. Sheet 3 contains calculations of summary statistics of sequencing utilizing data in Sheet 1.

Table S2 Caption

Table S2. Complete results of GO enrichment analysis. Results of GO enrichment analyses from gProfiler for all candidate genes resulting from our analysis. Includes database source, GO term name, GO term ID, and adjusted p-values amongst other parameters.

Table S3 Caption

Table S3. Data from SnpEff analysis of candidate genes. Data representing gene names with HGNC and Ensembl IDs. Additional columns represent variant types including but not limited to 3 prime UTR, 5 prime UTR, missense coding, nonsense coding, intron variants.
